# The small hive beetle’s capacity to disperse over long distances by flight

**DOI:** 10.1038/s41598-024-65434-1

**Published:** 2024-06-27

**Authors:** Bram Cornelissen, James D. Ellis, Gerrit Gort, Marc Hendriks, Joop J. A. van Loon, Charles J. Stuhl, Peter Neumann

**Affiliations:** 1 Netherlands Institute for Vectors, Invasive Plants and Plant Health (NIVIP), National Plant Protection Organization (NPPO), Netherlands Food and Consumer Product Safety Authority (NVWA), Geertjesweg 15, 6706 EA Wageningen, The Netherlands; 2 Honey Bee Research and Extension Laboratory, Entomology and Nematology Department, University of Florida, Gainesville, FL 32611 USA; 3 Biometris, Wageningen University & Research, P.O. Box 16, 6700 AA Wageningen, The Netherlands; 4 Wageningen Plant Research, Wageningen University and Research, P.O. Box 16, 6700 AA Wageningen, The Netherlands; 5 Laboratory of Entomology, Wageningen University & Research, P.O. Box 16, 6700 AA Wageningen, The Netherlands; 6 Center for Medical, Agricultural and Veterinary Entomology, USDA-ARS, Gainesville, FL 32608 USA; 7 Vetsuisse Faculty, Institute of Bee Health, University of Bern, Schwarzenburgstrasse 161, CH-3097 Bern, Switzerland

**Keywords:** *Aethina tumida*, *Apis mellifera*, Dispersal, Insect Flight, Invasive species, Biological invasion, Invasive species, Entomology

## Abstract

The spread of invasive species often follows a jump-dispersal pattern. While jumps are typically fostered by humans, local dispersal can occur due to the specific traits of a species, which are often poorly understood. This holds true for small hive beetles (*Aethina tumida*), which are parasites of social bee colonies native to sub-Saharan Africa. They have become a widespread invasive species. In 2017, a mark-release-recapture experiment was conducted in six replicates (A–F) using laboratory reared, dye-fed adults (N = 15,690). Honey bee colonies were used to attract flying small hive beetles at fixed spatial intervals from a central release point. Small hive beetles were recaptured (N = 770) at a maximum distance of 3.2 km after 24 h and 12 km after 1 week. Most small hive beetles were collected closest to the release point at 0 m (76%, replicate A) and 50 m (52%, replicates B to F). Temperature and wind deviation had significant effects on dispersal, with more small hive beetles being recaptured when temperatures were high (GLMM: slope = 0.99, SE = 0.17, Z = 5.72, *P* < 0.001) and confirming the role of wind for odour modulated dispersal of flying insects (GLMM: slope = − 0.39, SE = 0.14, Z = − 2.90, *P* = 0.004). Our findings show that the small hive beetles is capable of long-distance flights, and highlights the need to understand species specific traits to be considered for monitoring and mitigation efforts regarding invasive alien species.

## Introduction

Due to increased global trade, biological invasions have increased exponentially from the Middle Ages onwards^1,2^. The number of recorded invasive aliens species has accelerated during the past decades with a global doubling time of 14 years^3^. Invasive alien species affect the environment and impact society, for instance by outcompeting or parasitizing native species, and by vectoring zoonoses, transmittable to humans^4–6^. Invasive insect species alone are associated with an annual cost of US$ 70 billion globally and US$6.9 billion for the US^7^. A successful invasion hinges on the ability of a species to survive and reproduce in a newly found habitat and understanding what mechanisms make these few species successful is important to help mitigate their impact^8^.

Since 1996, small hive beetles (SHB; *Aethina tumida* Murray, Coleoptera: Nitidulidae) have spread from their native range of sub-Saharan Africa to the Americas, Asia, Australia and Europe^9^. The SHB established populations on all these continents and global warming is predicted to result in further range expansion^10^. This parasite of bee nests utilizes its hosts for feeding, hiding and reproduction^11–13^. In particular, mass reproduction events with often thousands of larvae can cause severe damage to seemingly healthy honey bee colonies often leading to the full structural collapse of the entire nest^14^ within 2 weeks’ time^15^. Past biological invasions of SHB show a strong correlation between genetics and international honey bee wax trade data, thereby suggesting that commercial apicultural imports might constitute the main gateway for crossing biogeographical boundaries^16^. Furthermore, there is clear evidence that trade with queen cages, package bees and entire honey bee colonies had resulted in a number of non-intentional SHB introductions (reviewed by^13^). In the introduced ranges, migratory beekeeping has shown to be a key for local spread (reviewed by 15). Therefore, it seems obvious that human-mediated jump dispersal plays an important role in the outward spread of the SHB. But since adult SHBs do fly, active flight may also contribute to dispersal within the introduced ranges. However, very little is known about the flight and dispersal capacity of the SHB and its ability to find hosts and possibly conspecifics under field conditions^18^. The SHB is considered an able flyer with anecdotal reports suggesting a flight range of 10 km a day^19^ and the ability to detect colonies 13 to 16 km away^20^. SHB-free apiaries are readily (re)infested by SHBs in areas where they are considered to be well-established. Reinfestation events in these areas show a correlation with the density of colonies and mass-reproduction events^15,18^. Yet, there are no dedicated studies investigating flight capacity over distances of more than 200 m^21^.

The mobility of adult insects is influenced by environmental factors. Insects have a lower and upper temperature threshold for flight activity^22^ and other weather conditions, such as precipitation can affect flight performance, due to increased body mass or a delay of flight initiation. For SHB, the host and host finding is another factor of importance. SHBs are attracted to honey bee and bumble bee colony odours when exposed in laboratory and field assays^23,24^ and likely use these cues to detect host colonies, which they then invade^11,12,17^. Host-, but also mate-finding has been shown under field conditions with mark-release-recapture experiments for other flying Nitidulidae which use chemotaxis^25,26^. Such odour-modulated flight of insects can be affected by wind and temperature. For instance, host odour plumes are shaped and distorted by wind, requiring insects to apply host finding strategies^27,28^ and temperature can affect the mobility of flying insects^22^. Moreover, intrinsic characteristics of individuals such as size, sex, age and physiological development have all been shown to affect the flight capacity and dispersal of insects^29,30^. Larger mountain pine beetles (*Dendroctonus ponderosae*) fly longer and farther than smaller individuals^31^ and mated *Arhopalus rusticus* beetles fly farther than unmated ones^32^. Also *A. rusticus* females, which are bigger than males, were stronger flyers than males. To what extent this applies to SHBs under field conditions is currently unknown. This information would be relevant for understanding dispersal and the invasion biology of the SHB. It would support contingency plans aimed at preventing or eliminating introductions of this beetle pest^33^. Honey bee colonies are used as sentinels for early detection monitoring of SHB introductions, for instance near sea ports^34,35^. However, there is no knowledge of the efficacy of such measures, or the spatial interval required for effective monitoring purposes.

To better understand the dispersal of the SHB by flight, we performed a mark-release-recapture experiment under field conditions in Florida, where SHB is well-established as an invasive alien species. We further define “dispersal” here as the capacity of adult SHB to fly, thereby possibly crossing so far non-determined long distances and contributing to their spread in the invasive ranges. We hypothesized that SHBs would fly further than the currently known distance of 200 m and that temperature, wind and humidity will affect SHB dispersal similar to other insects. Warm temperatures and humid conditions might lead to longer distances flown, while wind direction is expected to affect flight direction. Furthermore, we hypothesized that SHBs decrease in body mass due to the energy expenditure during flight and that body weight of individual beetles will therefore decrease as flight distance increases.

## Material & methods

Between May and December 2017, an experiment was performed at the Plant Science and Research and Education unit, located in Citra, FL. (29°24′30.7″N 82°10′15.8″W), where 32 *A. mellifera* colonies of mixed European origin were housed in five-frame nucleus Langstroth hives and placed in the field (29°24′30.7″N 82°10′15.8″W) to attract SHBs. Colonies contained four frames covered with bees and three frames with brood, with the remaining frames containing honey and pollen. The colonies were placed on pallets and sheltered underneath a white shade cover, because free-flying SHB are known to prefer shaded colonies over sun-exposed ones^36^. All colonies were fed with sugar water (saccharose, 40%, mass ratio) ad libitum. If found queenless during inspection, colonies were requeened or replaced with new nucleus colonies of similar composition within 2 weeks.

The experiment was performed once in May (replicate A) and five times between October and December (replicates B to F). For replicate A, 32 colonies were used between 22 and 25 May 2017. Single colonies were placed at 50, 100, 200 and 400 m in all wind directions (N, E, S, W) and three colonies each spread out on a North–South alignment at 800, 1200, 1600, 2400 and 3200 m eastward from the release point. One colony was placed right at the release point at 0 m (Supplementary Information File S1a).

On 25 September 2017, colonies were again placed at the same locations for replicates B to D as for replicate A, but no colony was placed at the release point. Due to a hurricane, the southernmost recapture point at 2400 m was flooded and therefore moved east by 100 m to a distance of 2500 m from the release point. For replicates E and F, a mirrored westward orientation layout was used with no colonies at 1200 m, but two at 1600 m and additional four nucleus colonies at 3600 m from the release point (Supplementary Information File S1b). In order to get information on dispersal over distances exceeding 3.6 km, two additional non-experimental apiaries away were included during replicates D to F. Depending on the location of the release point for replicate D, vs. E and F, these apiaries were located in Island Grove (N = 2 ten-frame honey bee colonies) at 5.9 and 5.1 km respectively and in Lochloosa (N = 12 ten-frame honey bee colonies) at 13.6 and 12.0 km respectively (Supplementary Information File S1c).

Experimental SHBs (*Aethina tumida*, Murray, Coleoptera: Nitidulidae) were reared in the USDA-ARS laboratory in Gainesville, Florida, USA^37^. Emerged adults were collected from the pupation containers twice a week and maintained in mixed-sex groups of 250 each in 3 L plastic containers with sugar water (saccharose, 40%, mass ratio) ad libitum at 25 °C under constant darkness. Sugar water was refreshed and dead specimen were removed twice a week. The average age of adult SHBs was 16 days old (SD 5.4) at the time of release and can therefore be regarded as sexual mature^38^ and likely mated.

In order to mark *en masse*, SHBs were fed three consecutive days a sucrose solution 40% with Rhodamine B (Sigma-Aldrich) dye (1%) using cotton dental rolls dipped in the solution for ten minutes. Rhodamine B is a dye commonly used for internal marking of insects^39,40^. The drenched dental rolls were placed in a partially covered Petri dish to limit evaporation, but still allowing access by SHBs. Pilot studies indicated that the survival rate of dye fed SHBs eleven days post-feeding was 100% (n = 11), similar to SHBs fed only sugar water (40% solution; n = 11). In the laboratory, the dye could be observed in SHBs for 15 days after feeding (data not shown). In order to establish pre-release body mass and sex ratio for each replicate, three SHBs (in total 31 to 64 per replicate) were collected from every feeding container prior to each release, then sexed, weighed and squashed to check if the dye could be observed. In all but one sampled SHB (n = 230), the dye could be observed in the abdomen.

Dye-marked SHBs were released for replicates A to F (Table 1) with a minimum of 14 days between release dates. The SHBs were transferred to the release point in plastic containers, placed together on a pallet underneath a white shade and rain cover and then opened. The date, time and temperature at the time of release are given in Table 1. SHBs were released in dry weather conditions only. The following day, the SHBs remaining in the containers were counted and removed. This constituted less than 1% of the total number of SHBs released. SHBs were recaptured from the colonies by visually screening^41,42^ one, two, four and seven or eight days after release. Due to unfavourable weather conditions, day four inspections for replicate E and F were skipped. Sampled SHBs were kept in vials within a cool box containing ice packs and transferred into a fridge until further processing. Within 24 h after collection, all captured SHBs were weighed, sexed, and then frozen at -20 °C until they were checked for dye marking via squashing them on a filter paper (Whatman, ⊘ = 10 cm). SHBs were considered positive, when red staining of the gut content could be observed with the eye or a stereomicroscope (Leica, M205C, Wetzlar, Germany). As Rhodamine B has fluorescent properties, we also checked samples under UV light (Fotodyne, RM-0026–0, Heartland, Wisconsin). An unmarked control SHB was used each time for comparison with autofluorescence naturally present in SHB tissue. Fluorescent staining of dye-marked SHBs was recognisable as red to yellow-greenish coloured compared to faint green for autofluorescence (Supplementary Information File S1d).

**Table 1:** Mark-release-recapture of adult small hive beetles (SHB, *Aethina tumida*).

Replicate	Date	Temperature °C (min–max)	Released	Recaptured	Recapture rate	Unmarked	Total
A	29 May 2017	28.3 (23.9–34.5)	3105	145 (605)	4.7% (19.5%)	161 (182)	306 (787)
B	9 Oct 2017	27.7 (24.5–33.0)	889	29	3.26%	78	107
C	23 Oct 2017	20.9 (15.9–25.1)	3573	22	0.62%	19	41
D	6 Nov 2017	21.7 (17.3–28.3)	3079	41	1.33%	110	151
E	20 Nov 2017	15.6 (8.1–23.5)	1619	22	1.36%	45	67
F	4 Dec 2017	18.8 (14.7–24.7)	3425	51	1.49%	35	86
Total			15,690	310	1.98%	448	758

The replicates, dates of release, minimum, maximum and average temperatures (°C) for 24 h after release, the number of released and recaptured marked SHB, recapture rates and the number of captured unmarked SHB are shown. For replicate A, the numbers between brackets show the results when the (re)captures at the release point are included. SHBs (n = 2) that flew away during hive inspections, and unmarked SHBs (n = 144) that collected after colonies were replaced, are not included in the table.

Weather data for the experimental site were obtained from the Florida Automated Weather Network (https://fawn.ifas.ufl.edu/) and collected at 15 min intervals: temperature (°C, 2 m above ground), relative humidity (%), rainfall (cm), wind speed (km/h) and wind direction (0° to 360°, collected 10 m above ground). Averages were calculated for the actual time intervals between release and recapture on day one and for the interval between recaptures for day two, four, seven and eight for replicates B to F. For replicate A, the exact time of recapture was not recorded and therefore 24 h averages prior to noon on recapture days were calculated except for day one. Since SHBs were released at 17.30 the previous evening, averages for day one entailed an 18.5 h interval. Data on wind direction were converted from degrees to eight units of 45° representing the cardinal and intercardinal directions. Furthermore, wind deviation (units of 45°) was calculated by relating the position of recapture locations relative to the release point to the wind direction. Minimum and maximum values for wind deviation were 0 (wind blowing from same direction as position of recapture location relative to release point), and 2 (wind blowing from opposite direction).

### Statistical analyses

For replicate A, descriptive statistics were calculated only and due to the different setup, this replicate was omitted from further statistical analyses. For replicate B to F, two datasets were analysed (Supplementary Information File S2). The first constituted of the number of recaptured dyed and undyed SHBs per nucleus colony for the given time and location during the experiment. The second dataset contained data related to the characteristics (sex and body mass) of pre-release and recaptured dyed individual SHBs. All analyses were performed using R software (R version 3.6.1^43^). First, a Generalized Linear Mixed Model (GLMM) with a negative binomial distribution and log link function was fitted with the count of recaptured marked SHBs as the response variable. The following fixed explanatory variables were included in the model. We used the number of released SHBs (log) and time since the last observation (log) as offset variables. We used distance from the release point (log), time since release (log) as regressors for space and time. Also, we included colony position expressed as the cardinal direction in relation to the release point as well as the location of the release point. The latter reflects the alternate location of the release point and colonies for replicate B to D, compared to E and F (see Supplementary Information File S1a-b). The climate variables temperature, relative humidity, rainfall, wind speed (all standardized variables, i.e. with mean zero and standard deviation of one) and wind deviation were also included in the model. Explanatory variables with random effects were replicate and hive location, to account for the multiple observations from the same physical location. Additionally, we used function dredge from R package MuMIn^44^ which fits all-possible subsets, ordering the resulting model fits by (corrected) Akaike’s Information Criterion. We wanted to know if males and females responded differently. Therefore, we extended the model set up for marked SHBs with main effects of sex and interactions of all other regressors with sex. Because of data sparsity (only 36 males were recaptured) we are cautious about this approach and look at it as a secondary analysis.

We ran a similar model (GLMM, negative binomial distribution) for SHBs we captured that were unmarked, and were thus not released. These represent free-flying SHBs that were entering the colonies and were collected under the same environmental circumstances as the marked SHBs. The model for unmarked SHBs differed in that we included only climate variables and time since last observation as an offset. We excluded captures on time points when colonies had been replaced (n = 6), as these colonies were not checked for SHBs prior to use. For fitting the negative binomial GLMMs, we applied the function glmmTMB from package glmmTMB^45^.

The second dataset was used to analyse differences in body mass and sex of recaptured marked SHBs and to compare them with those of marked SHBs that were sampled prior to release. Therefore, we fitted a GLMM for the body mass of individual beetles, as measured before release and after recapture, assuming a normal distribution for the body mass, fixed effects for source (pre-release/recaptured) and sex, and random effects for replicate and location. Hereafter, we modelled, for recaptured SHBs only, the body mass with fixed effects for distance from the release point (log), day since release, sex, and climate variables (temperature, relative humidity, rainfall and wind speed, and random effects for replicate and hive location). For this, again a GLMM was used, assuming a normal distribution for body mass. To fit these models, we applied function glmmTMB employing the ability to model both means and variability simultaneously, as we found considerable differences in variability of SHB body mass between replicates and between pre-release and recaptured groups. To check model assumptions, we made residual plots and checks using R-package DHARMa^46^. In none of the analyses the residual plots and checks showed remarkable deviations from the model assumptions. The statistical analyses can be found in Supplementary Information File S3.

## Results

### Observations replicate A

**Figure 1:**
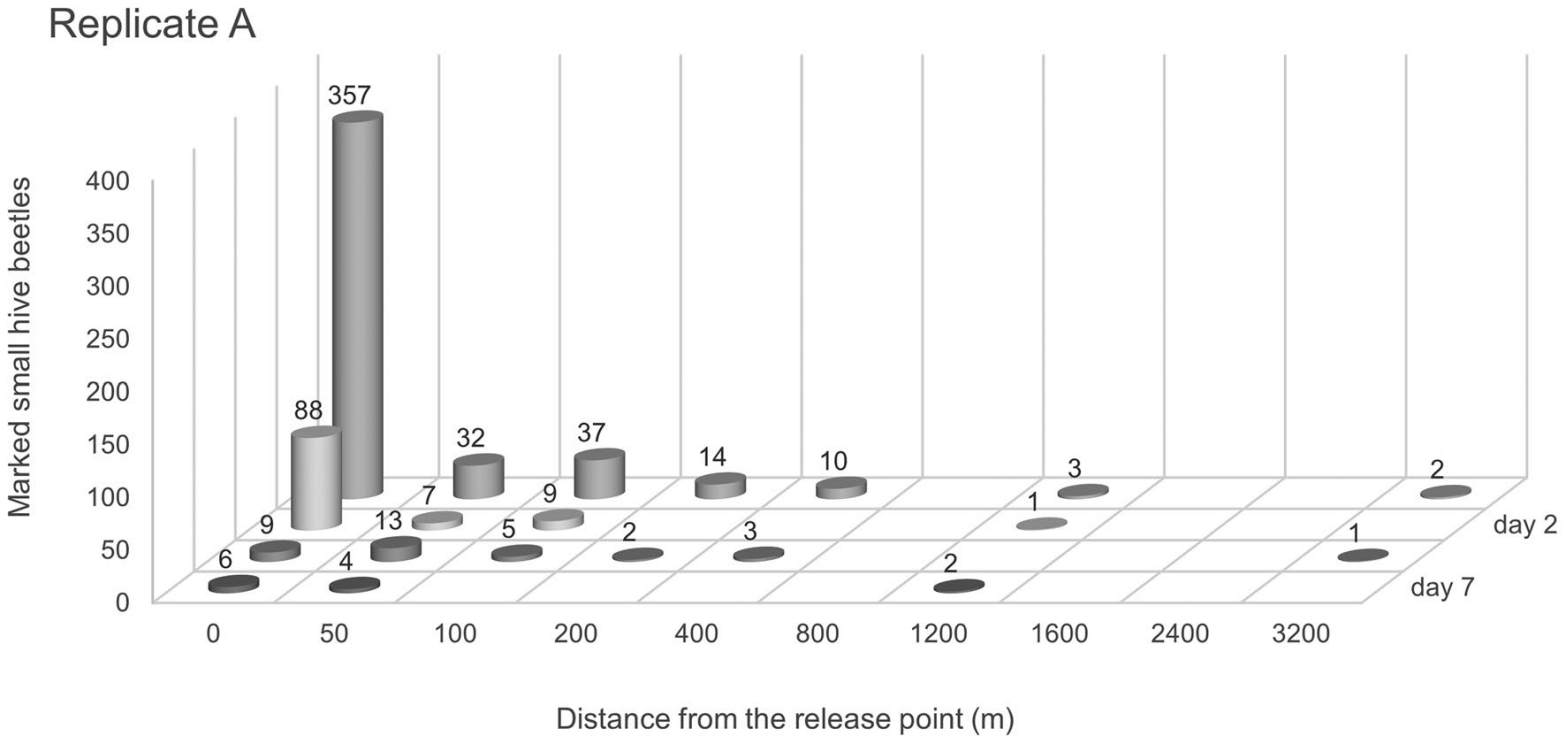
Recaptured small hive beetles during replicate (**A**). Marked small hive beetles (SHB, *Aethina tumida*) recaptured during replicate (**A**) (May–June 2017) in honey bee (*Apis mellifera*) colonies. The x-axis shows the distance in meters in all directions from the release point at which SHB were recaptured. The y-axis shows the number of marked SHBs recaptured and the Z-axis at which day after release the SHB were recaptured.

The weather conditions during replicate A are shown in Table 2. A total of 605 of 3105 marked SHBs were recaptured over the course of a week, of which 460 were found in the colony that was placed at the release point (0 m, Fig. 1). Another 145 marked SHBs were recaptured up to 3200 m from the release point, of which 54 SHBs were found at a distance of 50 m from the release point. Within a day after release, two marked SHB females were recovered from a colony at a distance of 3200 m from the release point.

**Table 2:** Weather variables, temperature, relative humidity, rain fall and wind speed, recorded at the Citra experimental research facility, Citra, Florida, during mark, release, recapture experiments of *Aethina tumida* in 2017.

Replicate	Period (from / to)	Tavg (C)	Tmin (C)	Tmax (C)	Rel hum (%)	Rain (mm)	Wind (km/h)
a	29 May 2017 / 05 June 2017	25,8	20,9	35,1	81	53,3	6,6
b	9 Oct 2017 / 16 Oct 2017	25,8	19,9	33,5	83	26,4	6,5
c	23 Oct 2017 / 30 Oct 2017	17,0	3,7	28,6	72	5,8	7,5
d	6 Nov 2017 / 13 Nov 2017	19,9	12,9	28,8	85	1,8	6,5
e	20 Nov 2017 / 27 Nov 2017	16,3	7,3	26,0	86	74,7	5,9
f	04 Dec 2017 / 11 Dec 2017	12,9	− 0,4	27,4	86	40,6	7,0

For temperature average, minimum and maximum recorded values are given in degrees Celsius, for relative humidity average percentages are given. For rain fall, the totals are shown in millimetres and average wind speed is given in kilometres per hour. The data shown was recorded every 15 min (n = 768 for each replicates) from the day of release up to and including 7 days after release.

### Observations replicate B to F

Weather conditions for replicate B to F are given in Table 2. During replicates B to F, 598 SHB were collected of which 165 (out of 12,585 released) were recaptured marked SHB (Table 1). Two SHB flew off during colony inspection and could not be assessed for dye markings. The remainder (n = 431) were unmarked and represented free-flying SHB (n = 287) that entered colonies during observations and unmarked SHB (n = 144) that were found in replacement colonies during the subsequent observation. Recapture rates varied between 0.62% to 3.26% per replicate.

### Dredge analysis

Dredge results show that some explanatory variables are always recurring in a selected subset of models with marked SHB as target variable (Table 3). These include day since release, distance, orientation, temperature, wind deviation and the offset variables SHB released and time since last observation.

**Table 3:** Dredge results for GLMM with marked adult small hive beetles (SHB, *Aethina tumida*) counts as target variable for replicates B to F.

Model	a	b	c	d	e	f	g	h	i
Intercept	− 17.86	− 17.83	− 17.84	− 17.84	− 17.80	− 17.82	− 17.86	− 17.87	− 17.83
Direction			+		+	+			
Day since release (log)	− 0.53	− 0.64	− 0.53	− 0.59	− 0.64	− 0.61	− 0.54	− 0.52	− 0.64
Distance (log)	− 1.44	− 1.43	− 1.41	− 1.43	− 1.40	− 1.40	− 1.44	− 1.44	− 1.43
Orientation	+	+	+	+	+	+	+	+	+
Rain							0.03	0.01	
Relative Humidity	− 0.30	− 0.23	− 0.28		− 0.21		− 0.30	− 0.24	
Temperature	1.02	1.03	0.98	0.91	1.00	0.89	1.02	0.88	1.03
Wind speed	− 0.21		− 0.22				− 0.20		− 0.13
Wind Deviation	− 0.29	− 0.28	− 0.39	− 0.28	− 0.38	− 0.40	− 0.28	− 0.29	− 0.28
Offset # SHBs released (log)	+	+	+	+	+	+	+	+	+
Offset Time since last observation (log)	+	+	+	+	+	+	+	+	+
df	11	10	14	9	13	12	12	11	10
Log Likelihood	− 298.4	− 299.6	− 295.8	− 301.1	− 297.1	− 298.3	− 298.4	− 300.6	− 299.6
AIC	619.3	619.6	620.3	620.5	620.7	621.1	621.4	621.6	621.6
Delta	0.00	0.21	1.00	1.14	1.40	1.75	2.06	2.27	2.28

The table shows all possible subsets up to delta 2.5 for the resulting model fits by (corrected) Akaike’s Information Criterion (AIC). Either the value for the slope of (standardized) quantitative regressors is given, or inclusion ( +) for qualitative variables. A missing value indicates the variable is not part of the subset. The model with the lowest AIC value has the best fit.

### GLMM recaptured small hive beetles

**Figure 2:**
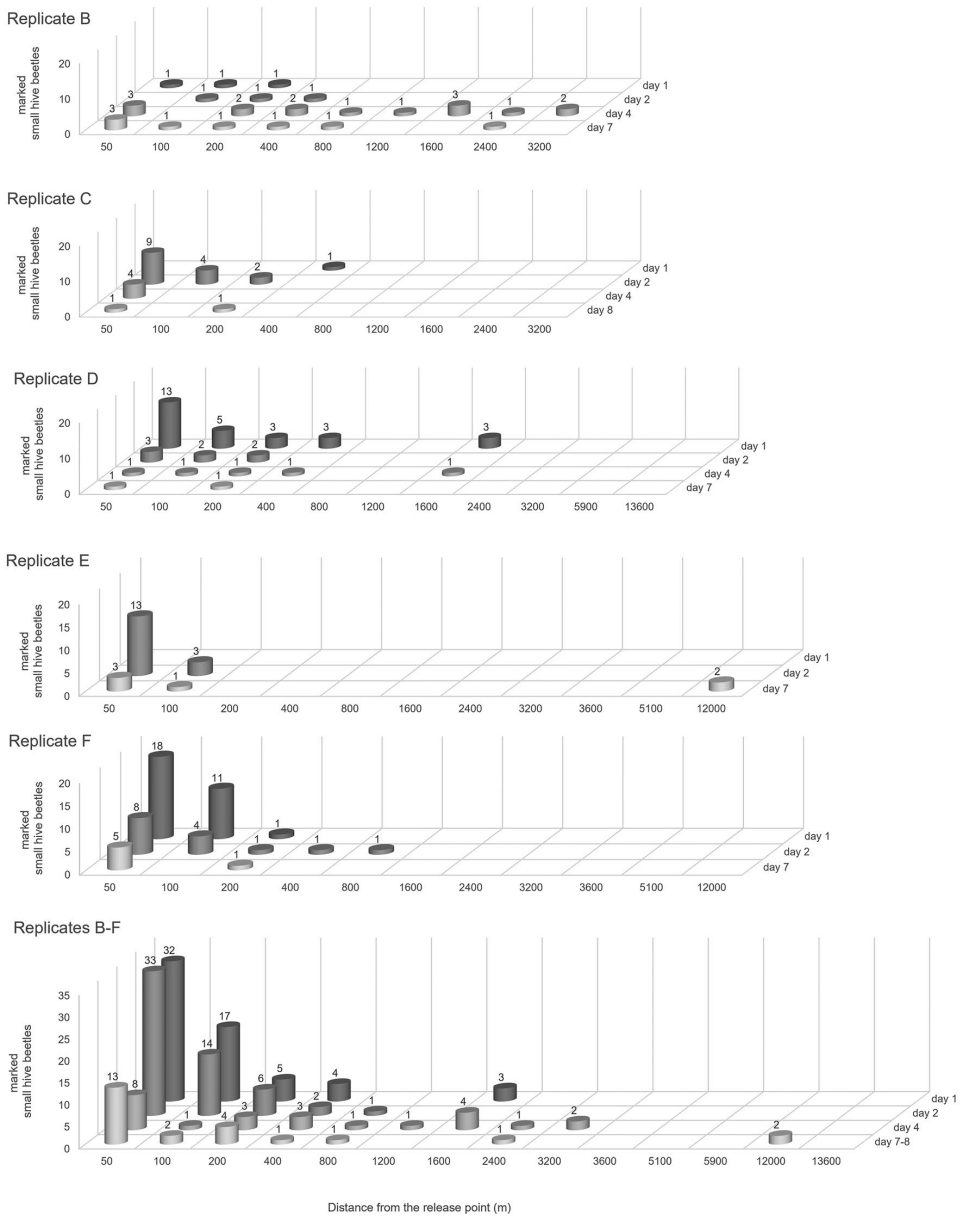
Recaptured small hive beetles during replicates (**B** to **F**). The number of marked recaptured adult small hive beetles (SHB, *Aethina tumida*) for replicates (**B** to **F**) and the total in honey bee (*Apis mellifera*) colonies recaptured in autumn. The x-axis shows the distance in meters in all directions from the release point at which SHB were recaptured. The y-axis shows the number of marked SHB recaptured and the Z-axis at which day after release the SHB were recaptured.

Fewer SHB were recaptured with an increasing distance from the release point (Fig. 2; GLMM: slope = − 1.41, SE = 0.17, Z = − 8.34, *P* < 0.001). The farthest distance from the release point where marked SHBs (n = 2) were recaptured was 12 km away, but most SHBs were caught at 50 m from the release point. Also, fewer SHB were recaptured as days since the release passed (slope = − 0.53, SE = 0.16, Z = − 3.35, *P* < 0.001).

Table 4a shows the results (Wald test) for the GLMM analysis of the number of recaptured SHBs. Temperature affected the recapture of marked SHBs (slope = 0.99, SE = 0.17, Z = 5.72, *P* < 0.001). More SHB were recaptured when temperatures were higher. No significant effects of either relative humidity, rain or wind speed were found (P > 0.05). However, a significant effect of wind deviation was found (slope = − 0.39, SE = 0.14, Z = − 2.90, *P* = 0.004). The higher the deviation, the lower the number of recaptured SHBs. For instance, if a nucleus colony was located to the north of the release point, more beetles were found when the wind was blowing from the north (deviation 0) compared to any other wind direction. A significant association was also found for the factor orientation (*P* = 0.025).

**Table 4:** Analysis of deviance for the GLMM analysis of the number of marked, recaptured small hive beetles (*Aethina tumida, SHB*).

Factors	Model a			Model b		
	Chi square	df	Pr(> Chisq)	Chi square	df	Pr(> Chisq)
Sex				8.28	1	0.004
Distance (log)	69.53	1	< 0.001	56.06	1	< 0.001
Day since release (log)	11.23	1	0.001	10.44	1	0.001
Direction	5.16	3	0.160	6.32	3	0.097
Orientation	5.04	1	0.025	3.35	1	0.067
Temperature	32.66	1	< 0.001	26.23	1	0.001
Relative humidity	3.45	1	0.063	0.77	1	0.380
Rain	0.00	1	0.962	0.07	1	0.788
Wind speed	2.45	1	0.117	2.49	1	0.115
Wind deviation	8.39	1	0.004	6.12	1	0.013
Sex : Distance (log)				1.41	1	0.235
Sex : Day since release (log)				0.38	1	0.539
Sex : Direction				2.37	3	0.500
Sex : Orientation				0.00	1	0.997
Sex : Temperature				0.01	1	0.940
Sex : Relative humidity				1.72	1	0.189
Sex : Rain				0.50	1	0.479
Sex : Wind speed				0.01	1	0.937
Sex : Wind deviation				0.02	1	0.875

Results are shown for the model without (a) and with (b) sex and interactions with sex as explanatory variables.

In the GLMM for marked beetles including factor sex, no significant interaction of any of the regressors with sex was found (Table 4b). Overall, an estimated 65% less males arrived than females (*P* = 0.004; comparison of sexes at average covariate values). In this model largely the same regressors were found to be important as found in the GLMM without sex: distance, day since release, temperature and wind deviation.

### GLMM unmarked small hive beetles

Unmarked SHB captures showed a partly different response to weather variables than marked ones. Similar to marked SHB, temperature had a significant effect, with a positive coefficient. No significant effect of relative humidity was observed, but rain fall and wind speed affected unmarked SHB captures. The more it rained, the fewer SHBs were captured (slope = − 0.30, SE = 0.12, Z = − 2.62, *P* = 0.009). Furthermore, high wind speeds led to lower captures of unmarked SHBs (slope = − 0.57, SE = 0.10, Z = − 5.75, *P* < 0.001).

### GLMM characteristics

Body mass of recaptured marked SHB was compared to that of marked SHBs that were collected prior to release. Female SHBs were on average 1.53 mg (SE 0.24) heavier than males, Z = 6.5, *P* < 0.001). No significant difference in mean body mass between the prelease and recaptured groups was found.

For recaptured SHBs only, marked females weighed 1.29 mg (SE 0.44) more than males (Z = 2.91, *P* = 0.004). No effect was found of distance from the release point at which marked SHBs were recaptured on body mass (slope = -0.48, SE = 0.28, Z = − 1.68 *P* = 0.093). However, body mass was related to rain (slope = 1.04. SE = 0.24, Z = 4.35, *P* < 0.001) and relative humidity (slope = 1.25, SE = 0.51, Z = 2.44, *P* = 0.015) with SHBs flying under rainy and humid conditions having significantly higher body mass than SHBs flying under dry weather conditions. No significant relation between temperature and wind speed with SHB body mass was found (temperature slope = 0.33, SE = 0.42, Z = 0.80, *P* = 0.43; wind speed slope = − 0.14, SE = 0.35, Z = − 0.38, *P* = 0.70).

## Discussion

Our data clearly show that flying SHB are able to cover a distance of at least 3.2 km a day and 12 km within a week. However, dispersal seems to be limited because the majority of SHBs were recaptured in colonies closest to the release point. Further, SHB flight is significantly promoted by warm temperature. SHBs, in particular females, prefer to fly upwind, likely using host cues to locate a colony^24^. The results obtained appear to be relevant for our understanding and mitigation of this invasive species.

In areas with high natural SHB infestation levels of honey bee colonies, such as in Florida, it is virtually impossible to establish SHB-free colonies. However, this is not of concern for answering our main research questions because we did mark the released SHB. Thus, due to the natural background infestation levels we were able to compare labelled vs. non-labelled SHB, which added naturally to our efforts. Neumann et al.^20^ performed a release- recapture experiment using unmarked, freshly emerged SHB and recaptured individuals in honey bee colonies at a distance of up to 200 m. No other studies have thus far recorded flight distance, although anecdotal reports suggested that SHB may fly several kilometres with flights over 10 km^19,20^. Systematic studies showed that apiaries were (re)infested after SHBs were removed from these apiaries, suggesting dispersal over several kilometres^15,18^. Similar results have been obtained using traps over shorter distances^47^. Reinfestation from unknown sources, however, could not be excluded and exact distances could not be ascertained. Potentially, the distance SHBs can travel by flight is even higher than what was found in this study. Indeed, even longer flights of other insects have been recorded with the assistance of wind (downwind) and vertical lift^48,49^. It is likely that in most cases, SHBs will limit flight as it is considered a costly and risky undertaking^29,48^. This is supported by the data showing that most recaptured SHBs were found closest to the release point, e.g. during replicate A (76%). SHB is attracted to the host^24,50–52^ and it is closely associated with *A. mellifera*^12^. SHBs can be considered an income breeder^53^, which needs to acquire resources for reproduction in the adult stage^38,54–56^. The sooner a colony is invaded, the sooner beetles get fed and reproduction can be initiated. This strategy also explains the significant negative correlation between the time since release and the number of recaptured females. Most females in the experiment were sexually mature, had likely already mated, and were subsequently in need of finding a host to provide resources for survival and reproduction. For males, no significant effect was found between time since release and the number recaptured, suggesting that males are more ambiguous towards host finding than females, which could possibly be aggravated if they have already mated. Suazo et al.^24^ showed that females were indeed more responsive to honey bee and bee-hive products than were males. This is further supported be the significant effect of wind deviation found in this study in favour of SHBs, in particular females, flying upwind towards a colony. Also, conspecific cues such as aggregation pheromones could play a role^57^. Although we removed SHBs from the colonies at regular intervals, it is likely that conspecific cues were still present and could be detected by released SHBs. To what extent conspecific cues play a role in dispersal is still largely unknown for SHBs^13^, yet very much relevant as the absence of congener cues during SHB invasions could affect the dispersal pattern. Such factors should be investigated in a similar fashion as the current study, for instance by controlling the number of SHB within the colony prior to release.

Nevertheless, a limited number of SHBs (n = 7) still performed long distance flights and ended up over three kilometres away from the release point due to unknown reasons. A migration syndrome^58^ of none-sexual mature individuals initially ignoring obvious host cues before settling^30^ may come into play. Mürrle and Neumann^59^ showed that adult SHBs can remain in the soil for up to 35 days and thus could have mated already well before taking flight. Others report just several days of dwelling in the soil^38,60,61^, in which case it is likely that not all emerging SHBs have yet reached sexual maturity, but are able to fly. Mustafa et al.^62^ showed that mating is most frequent when SHBs are aggregated and around 18 days old. The SHBs used in the experiment varied in age and had the opportunity to mate, but it is well possible that not all had mated. Therefore, the few long-distance migratory SHBs may have still been unmated. Controlled flight studies with SHBs of known age and physiological development are needed to deeper understand long range flight capacity and the possible relationship between dispersal and reproduction as in other insect species^32,63^.

Weather variables have been suggested to affect SHB flight and flight initiation^50^. This is supported by the dredge analysis in which temperature features in all shown models with best fits (Table 3). Significantly more SHBs were (re)captured under high temperatures compared to low ones within the local ranges of 10.7 °C to 26.6 °C (for replicates B to F). Night time temperatures dropped below 20 °C from replicate C onward during autumn, significantly reducing recaptures and thus apparently limiting SHB flight. . Our study provides a first indication of a lower and an upper temperature threshold for SHBs^22^, although it is likely that the range is narrower than the range of recorded temperatures. In particular, the lower threshold is likely to be higher than the lowest recorded temperature. Given the natural distribution of the SHB in Sub-Sahara Africa, it is unlikely that the SHB has developed any specific physiological cold-tolerance adaptation^64^. This notion is supported by the observation that the SHB has adapted a more general cold tolerance strategy by living within its hosts’ nest for most of its life stages^12^, specifically in the colony cluster^65^, where temperatures at the core of the cluster can be maintained at 25–31 °C even if ambient temperatures reach − 20°C^66^. Furthermore, other studies highlight the temperature dependency of other SHB traits such as oviposition and pupation^55,61,67^. Pupation occurs in the soil, where temperature is one of the key limiting factor for pupation success^10^. However, no such studies have been dedicated to flight performance. This is striking as temperature is a key factor explaining the activity and distribution of insects^64^ and dispersal is an important feature of invasion biology and distribution patterns of newly established populations^68^. In particular in temperate climates, the spread of the SHB might well be limited by the capacity to fly, due to low temperatures^69^. Likewise, it shows the opportunity at hand, when SHBs are introduced to (sub)tropical climates. SHB now occurs on all continents accept Antarctica from temperate to tropical climatic zones. Except for extreme low temperatures, the temperature range encountered by SHBs in our experiment broadly cover conditions in the invaded and native range. Overall, our study gives a robust account of the relation between temperature and SHB flight, but further experimental studies are needed to determine the thermal limits of SHB flight and the implications for invasion.

In our study, rain and relative humidity did not lead to a significant increase in the number of marked SHBs that were recaptured. Flight initiation of SHBs upon emergence from the soil is associated with rainy conditions preceding flight^38,50^ and colony infestation levels in savannah and forest conditions correlate with seasonal rains^36^, although SHB abundance in honey bee colonies in the US was not correlated to rainfall^70^. The outcome we obtained could in part be related to the experimental set up, as marked SHBs had already been taken from the soil and placed in plastic containers. Thus the conditions under which flight was initiated were not comparable to naturally occurring conditions. Also, no rain was observed in the hours prior to release.

Rainy conditions did lead to fewer captures of unmarked free flying SHBs, compared captures of the same during dry weather conditions. The differences with the results obtained for marked SHBs are likely explained by the different spatial scales and diffused distribution in the surrounding environment in relation to the locations where unmarked SHBs were captured. While rainfall could be a trigger or predictor of flight initiation and explain seasonal population dynamics, it is likely that rainfall as a weather condition in itself has an adverse effect on flight^71^. Similarly, wind speed affected the number of unmarked SHBs captured. Fewer were recaptured when wind speeds were high. Wind speed is known to affect upwind flight and flight initiation of other insect species with high wind speeds leading to less activity^72,73^.

Female SHB were heavier than males, which is in line with earlier research studies^54^. The average body mass of SHB did not change when they had flown, and no effect of the distance flown on body mass of SHB could be found for either sex. For insects, flight is an energetically costly process^29^, which in most cases leads to a decrease in body mass due to the exploitation of body components such as glycogen and lipids^74^. It is likely that the SHBs recaptured in the experiment lost body mass as a consequence of flying. However, the body mass loss could have been compensated by the intake of food in the hive soon after arrival^38^. Furthermore, our results showed that rain and relative humidity led to heavier SHB being recaptured. A possible explanation could be that heavier, more robust SHBs are better equipped to deal with such weather conditions than are SHBs of lower body mass.

Dispersal is a key factor of established and incipient invasive SHB populations and this study shows to what extent dispersal can play a role in the outward spread of SHBs. Most noteworthy is the distance SHB are able to cover, but also that SHBs tend to limit flight when they can. This study has also increased our understanding of the effect of weather variables on free-flying SHBs. The results of this study complement existing knowledge relevant for containing, mitigating and anticipating SHB as an invasive species, and as such, contributes to improve risk management along the whole chain^33^. Risk assessments prior to invasion or spread can include dispersal capacity under realistic weather and climate conditions. Outbreak management benefits by being able to better predict the dispersal rate and direction based on the local conditions. This will increase the effectiveness of contingency measures and decrease the chance of spread in the early stages of invasion. For instance, by taking current weather conditions into account. For instance, when maximum temperatures remain beneath the lower boundaries for flight that were observed in this study, the chance of dispersal is very low. This will increase the time window for adequate measures to be taken to prevent further spread.

As the number of introductions of invasive alien species grows exponentially, the need for biological and ecological base line information of species specific traits does so too^1,75^. Finally, it should be considered that invasive population can show a high genotypic variation depending on factors such as population size and origin^76^. Thus, variation in trait expression should be taken into account to explain differences in dispersal rates between invasive populations. This highlights the need to further investigate dispersal of the SHB and other invasive alien species.

### Supplementary Information

Supplementary Information 1.

Supplementary Information 2.

Supplementary Information 3.

## Data Availability

The datasets generated during and/or analysed during the current study are available in Supplementary Information File S3.
