# Supplementary material for: The small hive beetle’s capacity to disperse over long distances by flight
Source: Sci Rep. 2024 Jun 27;14:14859. doi: 10.1038/s41598-024-65434-1 (PMC11211503; doi:10.1038/s41598-024-65434-1)

The small hive beetle's capacity to disperse over long distances by flight

Bram Cornelissen, James D. Ellis, Gerrit Gort, Marc Hendriks, Joop J.A. van Loon, Charles J. Stuhl, Peter Neumann

Supplementary Information File S1

Supplementary Information File S1a. Map showing the spatial design of the mark release recapture experiment for replicates A-D at the Plant Science Research and Education Unit (Citra, Florida, US) experimental farm of the university of Florida. Indicated with a blue cross-sectioned circle is the release point at which location marked small hive beetles (SHB, *Aethina tumida*) were released. The location of honey bee colonies used to recapture SHBs are marked with a yellow diamond. Note that a honey bee colony was also present at the release point for replicate A. In the southern part of the prairie a blue arrow indicates the relocation of a recapture colony due to the flooding of the prairie in August 2017.

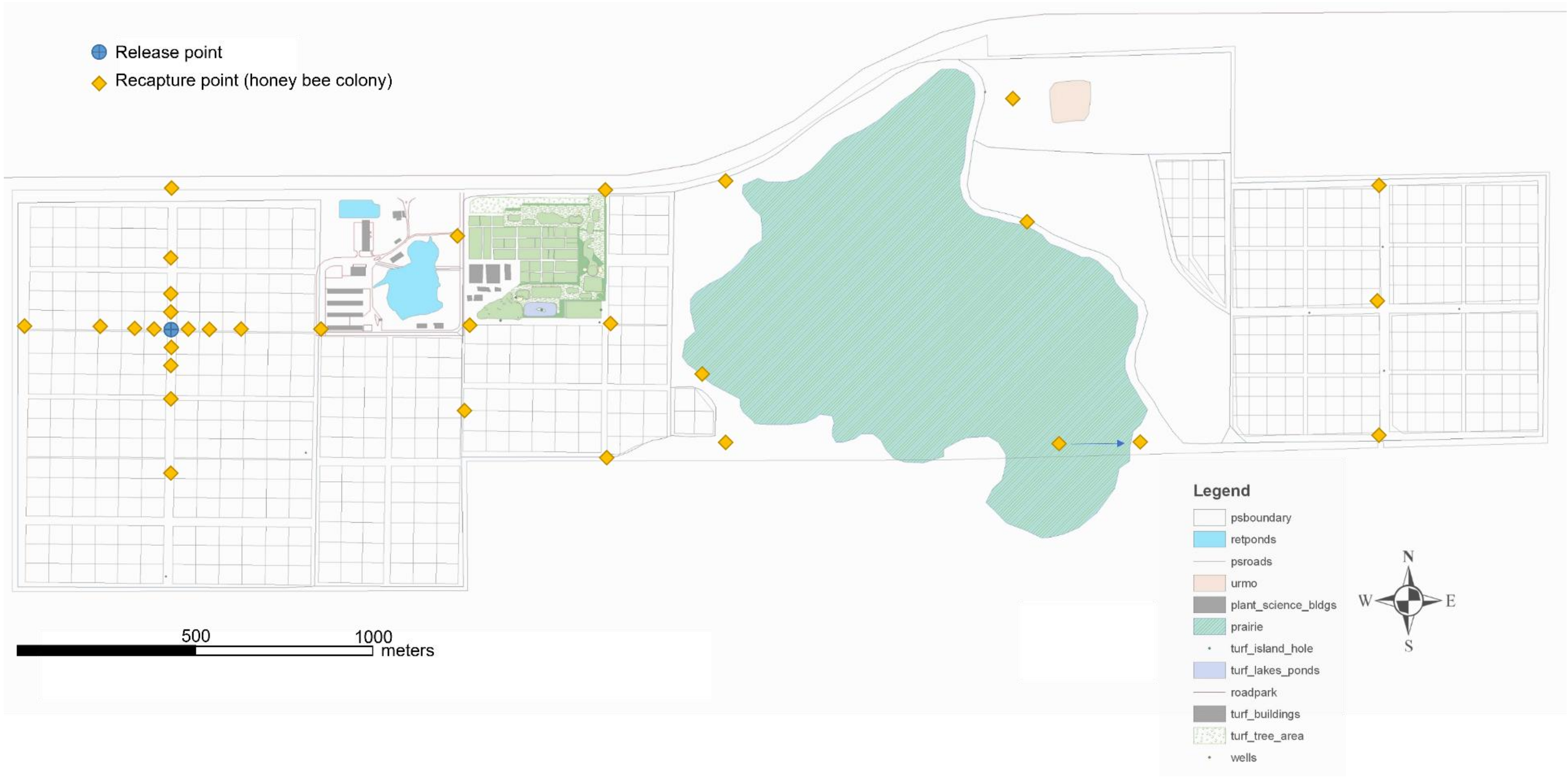

Supplementary Information File S1b. Map showing the spatial design of the mark release recapture experiment for replicates E and F at the Plant Science Research and Education Unit (Citra, Florida, US) experimental farm of the University of Florida. Indicated with a cross-sectioned blue circle is the release point at which location marked small hive beetles (SHB, *Aethina tumida*) were released. The location of honey bee colonies used to recapture SHBs are marked with a yellow diamond.

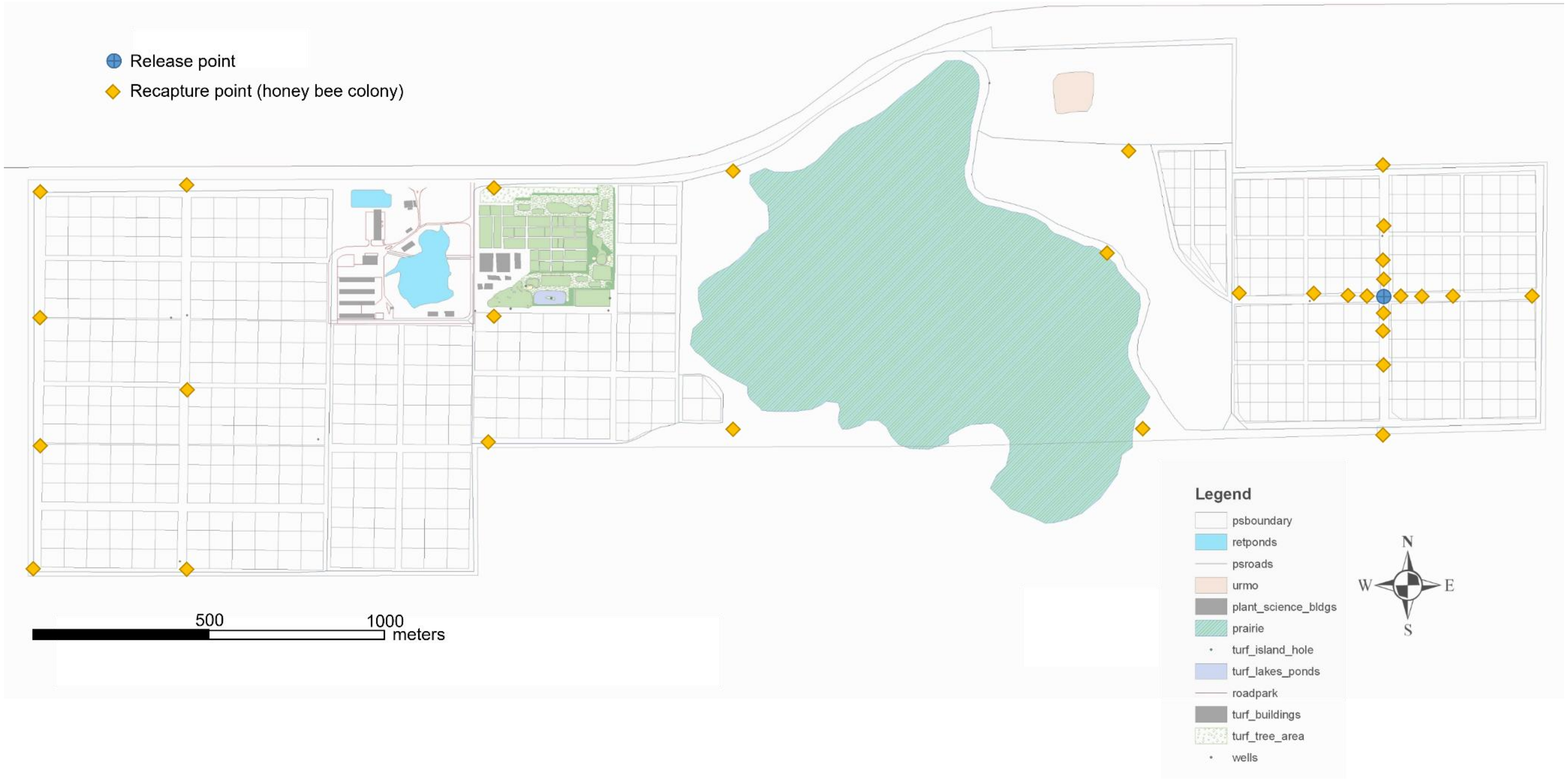

Supplementary Information File S1c. Map showing the location of the apiaries used for collecting small hive beetles at distances exceeding 3.6km. The orange triangles indicate the apiaries at (a) Island Grove and (b) Lochloosa. The location of the release points are indicated with a blue diamond: (1) release point for replicates A B, C and D, (2) release point for replicates E and F. Base map and data from OpenStreetMap and OpenStreetMap Foundation. ©Contributors, openstreetmap under licence (CCBY-AT 2.0).

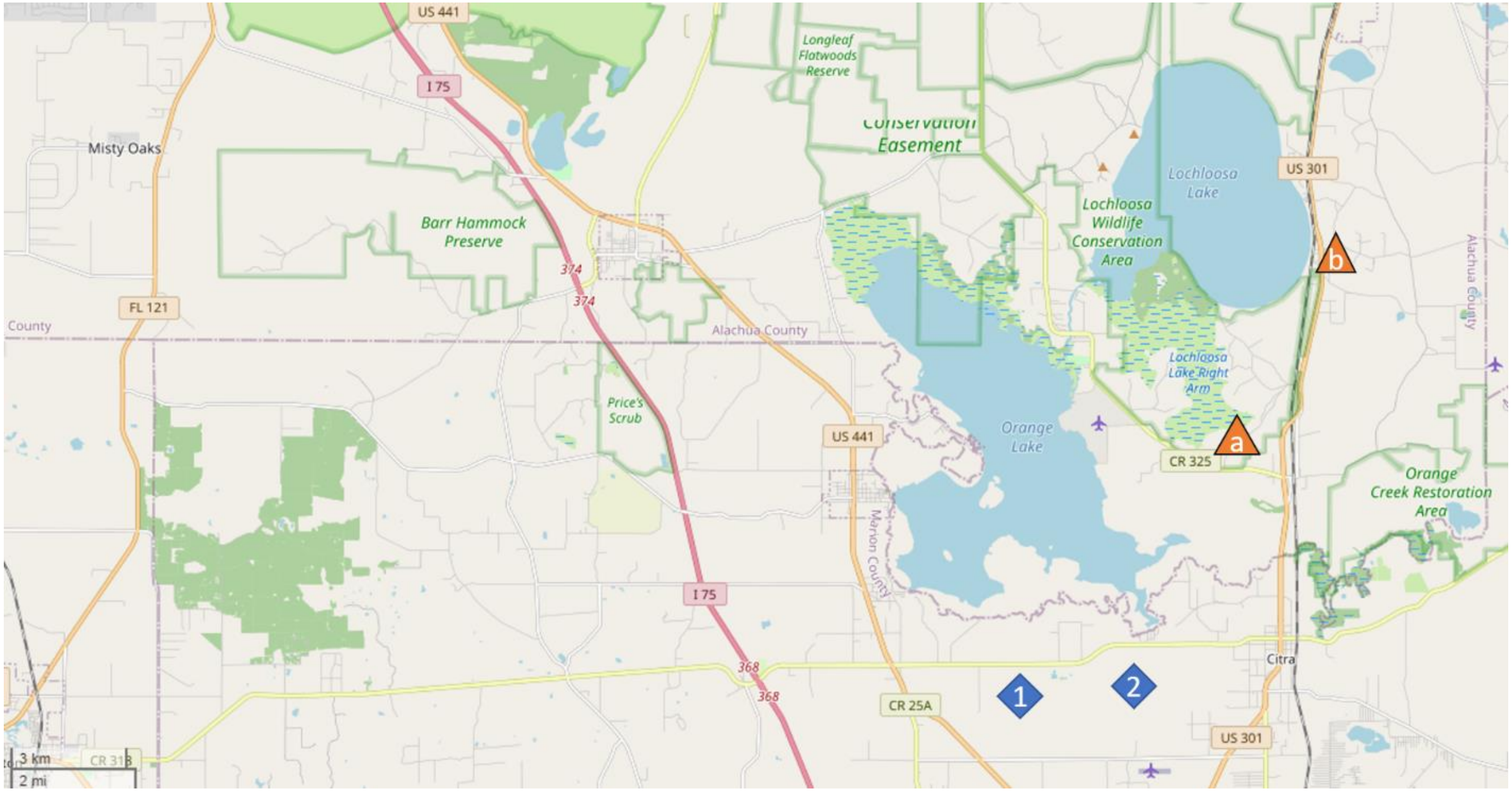

Supplementary Information File S1d. Small hive beetles (SHB, *Aethina tumida*), squashed on a Whatman filter for the purpose of establishing the presence of fed dye markings. SHBs were dye-fed with a sugar solution containing Rhodamine B. SHB '-C' is a negative control, SHB '12.1' is a field-captured unmarked SHB, and SHB '14.1' is a field captured dye-marked SHB. The left picture (A) shows the SHBs under laboratory tubular lighting. The right picture (B) shows SHBs under UV-light. Pictures taken with an iPhone S5 by J. Elmquist.

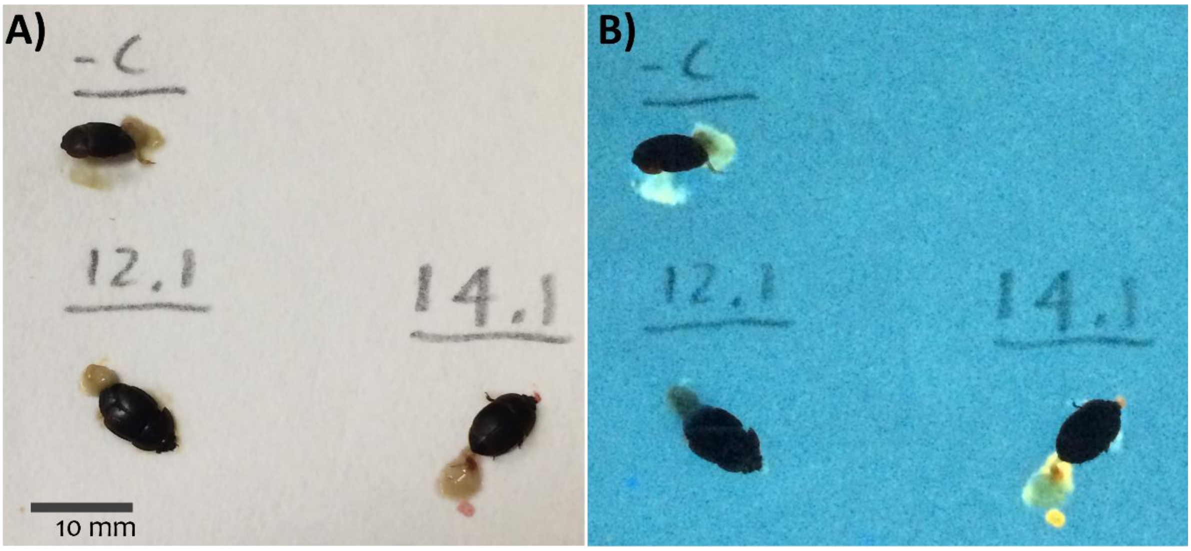

Supplement: Supplementary file 1 — Supplementary Information 1. [file 41598_2024_65434_MOESM1_ESM.pdf]
