# Supplementary material for: The small hive beetle’s capacity to disperse over long distances by flight
Source: Sci Rep. 2024 Jun 27;14:14859. doi: 10.1038/s41598-024-65434-1 (PMC11211503; doi:10.1038/s41598-024-65434-1)
Supplement: Supplementary file 3 — Supplementary Information 3. [file 41598_2024_65434_MOESM3_ESM.pdf]

The small hive beetle's capacity to disperse over long distances by flight

Bram Cornelissen, James D. Ellis, Gerrit Gort, Marc Hendriks, Joop J.A. van Loon, Charles J. Stuhl, Peter Neumann

Supplementary Information file S3

Study of dispersal and other properties of the Small Hive Beetle

Gerrit Gort and Bram Cornelissen

12-04-2024

Dispersal of Small Hive Beetles

Read hives dataset into R. Make sure that some variables are factors. Log-transform some regressors and offset variables. Standardize quantitative regressors by substraction of the mean and dividing by the standard deviation.

```
hives <- read.csv("HivesMRRSHB.csv")

hives$Replicate   <- as.factor(hives$Replicate)
hives$LOC.ID      <- as.factor(hives$LOC.ID)
hives$Dir         <- as.factor(hives$Dir)
hives$Orientation <- as.factor(hives$Orientation)

hives$logNrelease <- log(hives$SHB.RELEASED) # will become offset

# standardize (and log transform some) quantitative regressors
hives$TEMPs             <- scale(hives$TEMP)
hives$WSs               <- scale(hives$WS)
hives$RHs               <- scale(hives$RH)
hives$RAINs             <- scale(hives$RAIN)
hives$Dist.Ms           <- scale(hives$Dist.M)
hives$logDist.M         <- log10(hives$Dist.M)
hives$logDist.Ms        <- scale(hives$logDist.M)
hives$logDAY.Since.REL  <- log10(hives$DAY.Since.REL)
hives$logDAY.Since.RELs <- scale(hives$logDAY.Since.REL)

x1 <- as.numeric(hives$Dir)
x2 <- (0.5+hives$WDIR/2)
hives$WDdev <- pmin(rowSums(cbind(x1,-x2)) %% 4, rowSums(cbind(-x1,+x2)) %% 4)
# Variable WDdev is the deviation (units of 90 degrees) of wind direction from position of
# colony w.r.t. release point.
# E.g. DIR=1 (colony N of release point; WDIR=3 (wind from E); WDdev=1 (= 90 degr dev from colony pos).
# E.g. DIR=2 (colony E of release point; WDIR=3 (wind from E); WDdev=0 (= 0 degr dev from colony pos),
# meaning wind blows towards release point.
hives$WDdevs <- scale(hives$WDdev)

# t.since.last.OBS is defined as 0 for first obs, but it should be the time since release then.
hives$t.since.last.OBS2 <- hives$t.since.last.OBS
hives$t.since.last.OBS2[hives$t.since.last.OBS2==0] <-
  hives$t.since.release[hives$t.since.last.OBS2==0]
hives$logt.since.last.OBS2 <- log(hives$t.since.last.OBS2)

# some colonies have been replaced; the observations from that moment should be discarded.
Replaced <- (hives$REPLACED==1)
```

Totals counts and frequency tables of counts for hive dataset

```r
table(hives$SHB.TOTAL)
```

```
##
##   0   1   2   3   4   5   6   7   9  12  15  18  21  22  59
## 381 109  42  24  13  12   7   2   2   1   1   1   1   1   1
```

```r
sum(hives$SHB.TOTAL[hives$REPLACED != 1], na.rm=TRUE)
```

```
## [1] 451
```

```r
table(hives$SHBDYED)
```

```
##
##   0   1   2   3   4   5   6   7   8
## 506  56  18   8   3   2   2   1   1
```

```r
sum(hives$SHBDYED, na.rm=TRUE)
```

```
## [1] 165
```

```r
table(hives$SHBNODYE)
```

```
##
##   0   1   2   3   4   5   6   9  12  18  21  22  59
## 431 106  19  17   5   9   3   1   2   1   1   1   1
```

```r
sum(hives$SHBNODYE[hives$REPLACED != 1], na.rm=TRUE)
```

```
## [1] 287
```

Generalized linear mixed models for counts of recaptured beetles

Recaptured SHB: dispersal pattern and relation to diverse factors (without sex)

We analyze variable SHBDYED, i.e. the number of recaptured dyed beetles, which were released before. For this variable we start off with a negative binomial distribution (with log link function), because the count shows a skewed distribution, with lots of zeroes.

Explanatory variables with fixed effects:

Offsets: log(Nrelease) and log(time since last observation), because we expect that with twice as many released beetles, twice as many will be recaptured, and with doubling the waiting time until the next observation, also the number of recaptures will be (approximately) doubled.

Regressors related to space and time:

log(distance to release point) and log(time since release), because we expect that hives further away from the release point will attract less beetles, and as time passes by less and less beetles will be recaptured.

position of hive w.r.t. release point (factor Dir with four levels: North, East, South, West of release point), orientation (factor Orientation with two levels: replicates 6 and 7 have different orientation of hives compared to replicates 3, 4 and 5: release point was changed, and therefore position of hive w.r.t. release point changed.)

Regressors related to weather: Temperature, relative humidity, amount of rain, wind speed and wind direction deviation.

All quantitative regressors are standardized, i.e. with mean zero and standard devation 1.

For all (qualitative) factors we use the sum-to-zero restriction as parameterization.

Explanatory variables with random effects:

Replicate, but realize that aspects of replicates are already in fixed part of model (offset log(Nrelease) and factor Orientation)

LOC.ID: per hive-location combination "repeated" observations are obtained. These stretch over multiple replicates.

```
h <- hives[,c("SHBDYED", "SHBNODYE","logNrelease","logt.since.last.OBS2","logDist.Ms",
              "logDAY.Since.RELs","Dir","Orientation",
              "TEMPs","RHs","RAINs","WSs","WDdevs",
              "Replicate","LOC.ID")]

h <- na.omit(h)    # only cases without missing values are selected
dim(h)
```

```
## [1] 597  15
```

```
# Run GLMM
glmmTMBoD <- glmmTMB(SHBDYED ~ offset(logNrelease) + offset(logt.since.last.OBS2)
                     + logDist.Ms + logDAY.Since.RELs + Dir + Orientation
                     + TEMPs + RHs + RAINs + WSs + WDdevs
                     + (1 | Replicate) + (1 | LOC.ID),
                     contrasts=list(Dir=contr.sum, Orientation=contr.sum),
                     family=nbinom2, data=h)
summary(glmmTMBoD)
```

```
##  Family: nbinom2  ( log )
## Formula:
## SHBDYED ~ offset(logNrelease) + offset(logt.since.last.OBS2) +
##     logDist.Ms + logDAY.Since.RELs + Dir + Orientation + TEMPs +
##     RHs + RAINs + WSs + WDdevs + (1 | Replicate) + (1 | LOC.ID)
## Data: h
##
##      AIC      BIC   logLik deviance df.resid
##    623.1    689.0   -296.6    593.1      582
##
## Random effects:
##
## Conditional model:
##  Groups    Name        Variance  Std.Dev.
##  Replicate (Intercept) 6.192e-10 2.488e-05
##  LOC.ID    (Intercept) 2.504e-01 5.004e-01
## Number of obs: 597, groups:  Replicate, 5; LOC.ID, 76
##
## Dispersion parameter for nbinom2 family (): 1.09
##
## Conditional model:
```

```
##                      Estimate Std. Error z value Pr(>|z|)
## (Intercept)        -1.784e+01  2.112e-01  -84.49  < 2e-16
## logDist.Ms         -1.412e+00  1.680e-01   -8.41  < 2e-16
## logDAY.Since.RELs  -5.288e-01  1.576e-01   -3.36 0.000793
## Dir1                5.535e-01  2.462e-01    2.25 0.024557
## Dir2               -3.081e-01  2.433e-01   -1.27 0.205370
## Dir3               -2.386e-01  2.662e-01   -0.90 0.370049
## Orientation1       -4.002e-01  1.812e-01   -2.21 0.027243
## TEMPs               9.750e-01  1.707e-01    5.71 1.13e-08
## RHs                -2.712e-01  1.495e-01   -1.81 0.069725
## RAINs              -4.118e-05  1.696e-01    0.00 0.999806
## WSs                -2.195e-01  1.392e-01   -1.58 0.114899
## WDdevs             -3.866e-01  1.338e-01   -2.89 0.003845
```

```r
# Analysis of deviance table using Wald-tests
Anova(glmmTMBoD, type="III")
```

```
## Analysis of Deviance Table (Type III Wald chisquare tests)
##
## Response: SHBDYED
##                       Chisq Df Pr(>Chisq)
## (Intercept)       7139.0892  1  < 2.2e-16
## logDist.Ms          70.6647  1  < 2.2e-16
## logDAY.Since.RELs   11.2580  1  0.0007928
## Dir                  5.4307  3  0.1428449
## Orientation          4.8753  1  0.0272433
## TEMPs               32.6102  1  1.126e-08
## RHs                  3.2895  1  0.0697246
## RAINs                0.0000  1  0.9998063
## WSs                  2.4855  1  0.1148985
## WDdevs               8.3558  1  0.0038446
```

```r
# Analysis of deviance table using likelihood ratio tests
drop1(glmmTMBoD, test="Chisq")
```

```
## Single term deletions
##
## Model:
## SHBDYED ~ offset(logNrelease) + offset(logt.since.last.OBS2) +
##     logDist.Ms + logDAY.Since.RELs + Dir + Orientation + TEMPs +
##     RHs + RAINs + WSs + WDdevs + (1 | Replicate) + (1 | LOC.ID)
##                   Df    AIC    LRT  Pr(>Chi)
## <none>               623.13
## logDist.Ms         1 686.89 65.760 5.094e-16
## logDAY.Since.RELs  1 631.20 10.070  0.001507
## Dir                3 622.63  5.503  0.138487
## Orientation        1 625.55  4.422  0.035484
## TEMPs              1 641.27 20.140 7.199e-06
## RHs                1 624.39  3.258  0.071093
## RAINs              1 621.13  0.000  0.999793
## WSs                1 623.66  2.526  0.111953
## WDdevs             1 629.74  8.609  0.003345
```

```r
# Check model assumptions using functions from DHARMa library
simulationOutputD <- simulateResiduals(fittedModel = glmmTMBoD, n=1000)
plot(simulationOutputD)
```

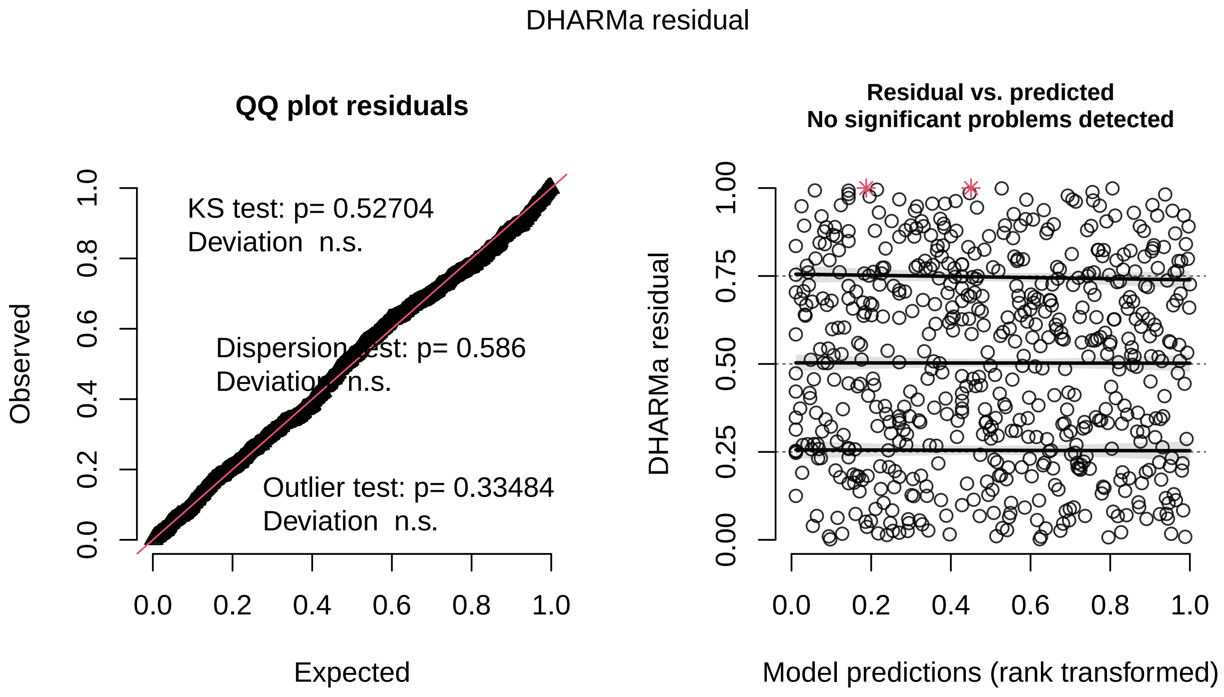


```r
# All possible subsets using dredge function.
# This procedure takes a lot of time; we read in previously obtained results.
# glmmTMBoD.dredge <- dredge(glmmTMBoD, tracT=2)
# saveRDS(glmmTMBoD.dredge, "glmmTMBoD-dredge.rds")
glmmTMBoD.dredge.read <- readRDS("glmmTMBoD-dredge.rds")

subset(glmmTMBoD.dredge.read, delta < 2.5)
```

```
## Global model call: glmmTMB(formula = SHBDYED ~ offset(logNrelease) + offset(logt.since.last.OBS2) +
##     logDist.Ms + logDAY.Since.RELs + Dir + Orientation + TEMPs +
##     RHs + RAINs + WSs + WDdevs + (1 | Replicate) + (1 | LOC.ID),
##     data = h, family = nbinom2, contrasts = list(Dir = contr.sum,
##         Orientation = contr.sum), ziformula = ~0, dispformula = ~1)
## ---
## Model selection table
##      cnd((Int)) dsp((Int)) cnd(Dir) cnd(lDA.Snc.REL) cnd(lgD.Ms) cnd(Orn)
## 2031     -17.86          +                 -0.5253      -1.440        +
## 1775     -17.83          +                 -0.6350      -1.433        +
## 2032     -17.84          +        +        -0.5310      -1.408        +
## 1743     -17.84          +                 -0.5940      -1.432        +
## 1776     -17.80          +        +        -0.6447      -1.398        +
## 1744     -17.82          +        +        -0.6085      -1.397        +
## 2047     -17.86          +                 -0.5366      -1.440        +
## 1999     -17.87          +                 -0.5183      -1.436        +
## 1791     -17.83          +                 -0.6408      -1.433        +
## NA
## NA.1
```

```
##      cnd(RAI) cnd(RHs) cnd(TEM) cnd(WDd) cnd(WSs) cnd(off(lgN))
## 2031           -0.2957   1.0150  -0.2856  -0.2094             +
## 1775           -0.2330   1.0270  -0.2830                      +
## 2032           -0.2788   0.9829  -0.3917  -0.2201             +
## 1743                     0.9129  -0.2844                      +
## 1776           -0.2131   0.9975  -0.3839                      +
## 1744                     0.8930  -0.3981                      +
## 2047   0.0278  -0.3037   1.0240  -0.2849  -0.2108             +
## 1999                     0.8829  -0.2867  -0.1302             +
## 1791   0.0138  -0.2366   1.0310  -0.2826                      +
## NA
## NA.1
##      cnd(off(lgt.snc.lst.OBS)) df   logLik  AICc delta
## 2031                         + 11 -298.447 619.3  0.00
## 1775                         + 10 -299.588 619.6  0.21
## 2032                         + 14 -295.813 620.3  1.00
## 1743                         +  9 -301.091 620.5  1.14
## 1776                         + 13 -297.058 620.7  1.40
## 1744                         + 12 -298.282 621.1  1.75
## 2047                         + 12 -298.433 621.4  2.06
## 1999                         + 10 -300.620 621.6  2.27
## 1791                         + 11 -299.585 621.6  2.28
## NA
## NA.1
## Models ranked by AICc(x)
## Random terms (all models):
##   cond(1 | Replicate), cond(1 | LOC.ID)
```

Recaptured SHB: dispersal pattern and relation to diverse factors including sex

Each original observation is split into one count for males and one count for females. Next, the resulting factor sex is included in the GLMM, also including its interactions with other explanatory variables.

```
# Analysis including sex

# Make dataframe long, with information on sexes
h <- hives[,c("SHBDYED", "SHBNODYE","D.M","D.F","logNrelease","logt.since.last.OBS2","logDist.Ms",
              "logDAY.Since.RELs","Dir","Orientation",
              "TEMPs","RHs","RAINs","WSs","WDdevs",
              "Replicate","LOC.ID")]

hA <- gather(h, sex, count, D.M:D.F, factor_key=TRUE)
levels(hA$sex) <- c("M","F")

o <- order(hA$LOC.ID, hA$Replicate, hA$logDist.Ms, hA$Dir, hA$logDAY.Since.RELs)
hA <- hA[o,]

hA <- na.omit(hA)    # only cases without missing values are selected
dim(hA)
```

```
## [1] 1194   17
```

```
glmmTMBoDA <- glmmTMB(count ~ offset(logNrelease) + offset(logt.since.last.OBS2)
                   + sex
                   + logDist.Ms + logDAY.Since.RELs + Dir + Orientation
```

```r
                   + TEMPs + RHs + RAINs + WSs + WDdevs
                   + logDist.Ms:sex + logDAY.Since.RELs:sex + Dir:sex + Orientation:sex
                   + TEMPs:sex + RHs:sex + RAINs:sex + WSs:sex + WDdevs:sex
                   + (1 | Replicate) + (1 | LOC.ID),
                   contrasts=list(sex=contr.sum, Dir=contr.sum, Orientation=contr.sum),
                   family=nbinom2,
                   data=hA)

summary(glmmTMBoDA)
```

```
##  Family: nbinom2  ( log )
## Formula:          count ~ offset(logNrelease) + offset(logt.since.last.OBS2) +  
##     sex + logDist.Ms + logDAY.Since.RELs + Dir + Orientation +  
##     TEMPs + RHs + RAINs + WSs + WDdevs + logDist.Ms:sex + logDAY.Since.RELs:sex +  
##     Dir:sex + Orientation:sex + TEMPs:sex + RHs:sex + RAINs:sex +  
##     WSs:sex + WDdevs:sex + (1 | Replicate) + (1 | LOC.ID)
## Data: hA
## 
##      AIC      BIC   logLik deviance df.resid 
##    768.2    905.5   -357.1    714.2     1167 
## 
## Random effects:
## 
## Conditional model:
##  Groups    Name        Variance  Std.Dev. 
##  Replicate (Intercept) 9.792e-10 3.129e-05
##  LOC.ID    (Intercept) 3.868e-01 6.219e-01
## Number of obs: 1194, groups:  Replicate, 5; LOC.ID, 76
## 
## Dispersion parameter for nbinom2 family (): 1.56 
## 
## Conditional model:
##                        Estimate Std. Error z value Pr(>|z|)    
## (Intercept)           -1.877e+01  2.289e-01  -82.04  < 2e-16
## sex1                  -5.194e-01  1.805e-01   -2.88  0.00401
## logDist.Ms            -1.325e+00  1.770e-01   -7.49 7.01e-14
## logDAY.Since.RELs     -5.420e-01  1.678e-01   -3.23  0.00124
## Dir1                   6.545e-01  2.662e-01    2.46  0.01394
## Dir2                  -2.754e-01  2.712e-01   -1.02  0.30980
## Dir3                  -1.521e-01  2.980e-01   -0.51  0.60975
## Orientation1          -3.488e-01  1.907e-01   -1.83  0.06732
## TEMPs                  9.210e-01  1.798e-01    5.12 3.03e-07
## RHs                   -1.523e-01  1.735e-01   -0.88  0.38025
## RAINs                 -4.884e-02  1.814e-01   -0.27  0.78775
## WSs                   -2.319e-01  1.469e-01   -1.58  0.11454
## WDdevs                -3.521e-01  1.423e-01   -2.47  0.01338
## sex1:logDist.Ms        1.666e-01  1.402e-01    1.19  0.23470
## sex1:logDAY.Since.RELs  1.028e-01  1.673e-01    0.61  0.53880
## sex1:Dir1              1.714e-01  1.891e-01    0.91  0.36471
## sex1:Dir2              1.067e-01  2.152e-01    0.50  0.61986
## sex1:Dir3              4.757e-02  2.135e-01    0.22  0.82370
## sex1:Orientation1     -5.713e-04  1.533e-01    0.00  0.99703
## sex1:TEMPs             1.355e-02  1.788e-01    0.08  0.93959
## sex1:RHs               2.276e-01  1.734e-01    1.31  0.18931
## sex1:RAINs            -1.288e-01  1.814e-01   -0.71  0.47760
## sex1:WSs               1.162e-02  1.466e-01    0.08  0.93683
```

```
## sex1:WDdevs             2.206e-02  1.399e-01    0.16  0.87466
```

```r
# Analysis of deviance table using Wald-tests
Anova(glmmTMBoDA, type="III")
```

```
## Analysis of Deviance Table (Type III Wald chisquare tests)
##
## Response: count
##                          Chisq Df Pr(>Chisq)
## (Intercept)          6730.0306  1  < 2.2e-16
## sex                     8.2804  1   0.004008
## logDist.Ms             56.0643  1  7.014e-14
## logDAY.Since.RELs      10.4370  1   0.001235
## Dir                     6.3227  3   0.096923
## Orientation             3.3471  1   0.067324
## TEMPs                  26.2335  1  3.025e-07
## RHs                     0.7699  1   0.380247
## RAINs                   0.0725  1   0.787749
## WSs                     2.4904  1   0.114541
## WDdevs                  6.1182  1   0.013379
## sex:logDist.Ms          1.4121  1   0.234700
## sex:logDAY.Since.RELs   0.3778  1   0.538796
## sex:Dir                 2.3657  3   0.500059
## sex:Orientation         0.0000  1   0.997027
## sex:TEMPs               0.0057  1   0.939591
## sex:RHs                 1.7229  1   0.189314
## sex:RAINs               0.5043  1   0.477602
## sex:WSs                 0.0063  1   0.936832
## sex:WDdevs              0.0249  1   0.874659
```

```r
# Check model assumptions using functions from DHARMa library
simulationOutputDA <- simulateResiduals(fittedModel = glmmTMBoDA, n=1000)
plot(simulationOutputDA)
```

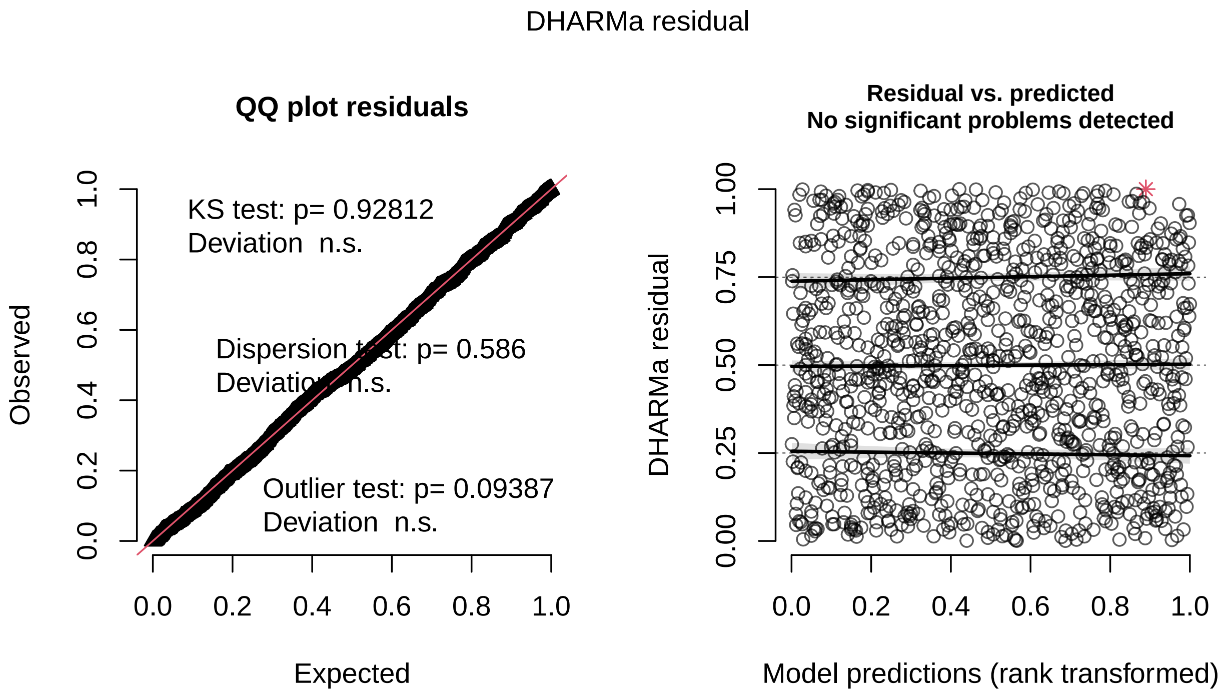


```r
# All possible subsets using dredge function.
# This procedure takes a lot of time (more than 4 days)
# We use previously obtained results.

# glmmTMBoDA.dredge <- dredge(glmmTMBoDA, trace=2)
# saveRDS(glmmTMBoDA.dredge, "glmmTMBoDA-dredge.rds")
glmmTMBoDA.dredge <- readRDS("glmmTMBoDA-dredge.rds")
# Print competing models at AICc value not further than 2 from best model.
subset(glmmTMBoDA.dredge, delta<2)
```

```
## Global model call: glmmTMB(formula = count ~ offset(logNrelease) + offset(logt.since.last.OBS2) +
##     sex + logDist.Ms + logDAY.Since.REL + Dir + Orientation +
##     TEMPs + RHs + RAINs + WSs + WDdev + logDist.Ms:sex + logDAY.Since.REL:sex +
##     Dir:sex + Orientation:sex + TEMPs:sex + RHs:sex + RAINs:sex +
##     WSs:sex + WDdev:sex + (1 | Replicate) + (1 | LOC.ID), data = hA,
##     family = nbinom2, contrasts = list(sex = contr.sum, Dir = contr.sum),
##     ziformula = ~0, dispformula = ~1)
## ---
## Model selection table
##          cnd((Int)) dsp((Int)) cnd(Dir) cnd(lDA.Snc.REL) cnd(lgD.Ms) cnd(Orn)
## 1577967      -17.86          +                    -1.743      -1.350        +
## 1573871      -17.93          +                    -1.741      -1.434        +
## 1577968      -17.64          +        +           -1.759      -1.311        +
## 1610735      -17.89          +                    -1.722      -1.346        +
## 1573872      -17.71          +        +           -1.757      -1.390        +
## 1606639      -17.96          +                    -1.723      -1.431        +
## 1610736      -17.66          +        +           -1.739      -1.308        +
## 1606640      -17.73          +        +           -1.738      -1.387        +
## 1577455      -17.73          +                    -2.059      -1.346        +
## 1639407      -17.94          +                    -1.755      -1.434        +
## 1573359      -17.80          +                    -2.057      -1.429        +
## 1643503      -17.88          +                    -1.752      -1.356        +
```

```
## 1639408     -17.72          +        +          -1.771      -1.391        +
## 1610223     -17.75          +                   -2.048      -1.342        +
## 1577456     -17.51          +        +          -2.086      -1.304        +
## 1573360     -17.58          +        +          -2.084      -1.383        +
## 1643504     -17.65          +        +          -1.768      -1.315        +
## 1577960     -17.39          +        +          -1.806      -1.350
## 1573864     -17.47          +        +          -1.803      -1.429
## 1606127     -17.82          +                   -2.046      -1.427        +
## 1672175     -17.97          +                   -1.734      -1.432        +
## 1840111     -17.87          +                   -1.740      -1.349        +
##         cnd(RHs) cnd(sex) cnd(TEM) cnd(WDd) cnd(WSs) cnd(lgD.Ms:sex)
## 1577967  -0.2857        +   0.9319  -0.4796  -0.2149               +
## 1573871  -0.2848        +   0.9281  -0.4792  -0.2144
## 1577968  -0.2778        +   0.9094  -0.6201  -0.2260               +
## 1610735  -0.1925        +   0.9344  -0.4817  -0.2193               +
## 1573872  -0.2771        +   0.9060  -0.6198  -0.2256
## 1606639  -0.1978        +   0.9304  -0.4806  -0.2182
## 1610736  -0.1846        +   0.9126  -0.6220  -0.2302               +
## 1606640  -0.1886        +   0.9090  -0.6226  -0.2295
## 1577455  -0.2288        +   0.9540  -0.4699                        +
## 1639407  -0.2836        +   0.9891  -0.4801  -0.2154
## 1573359  -0.2283        +   0.9503  -0.4695
## 1643503  -0.2842        +   0.9781  -0.4758  -0.2159               +
## 1639408  -0.2761        +   0.9655  -0.6192  -0.2264
## 1610223  -0.1431        +   0.9580  -0.4715                        +
## 1577456  -0.2181        +   0.9352  -0.5971                        +
## 1573360  -0.2178        +   0.9319  -0.5966
## 1643504  -0.2762        +   0.9561  -0.6169  -0.2268               +
## 1577960  -0.2068        +   0.7906  -0.6270  -0.2436               +
## 1573864  -0.2069        +   0.7888  -0.6266  -0.2432
## 1606127  -0.1478        +   0.9538  -0.4707
## 1672175  -0.2037        +   0.9857  -0.4813  -0.2171
## 1840111  -0.2847        +   0.9320  -0.4801  -0.2275               +
##         cnd(RHs:sex) cnd(sex:TEM) cnd(sex:WSs) cnd(off(lgN))
## 1577967                                                   +
## 1573871                                                   +
## 1577968                                                   +
## 1610735            +                                      +
## 1573872                                                   +
## 1606639            +                                      +
## 1610736            +                                      +
## 1606640            +                                      +
## 1577455                                                   +
## 1639407                         +                         +
## 1573359                                                   +
## 1643503                         +                         +
## 1639408                         +                         +
## 1610223            +                                      +
## 1577456                                                   +
## 1573360                                                   +
## 1643504                         +                         +
## 1577960                                                   +
## 1573864                                                   +
## 1606127            +                                      +
## 1672175            +            +                         +
## 1840111                                      +            +
##         cnd(off(lgt.snc.lst.OBS)) df   logLik  AICc delta weight
```

```
## 1577967                                  + 13 -362.696 751.7  0.00  0.077
## 1573871                                  + 12 -363.787 751.8  0.14  0.072
## 1577968                                  + 16 -359.804 752.1  0.37  0.064
## 1610735                                  + 14 -361.872 752.1  0.40  0.063
## 1573872                                  + 15 -360.860 752.1  0.43  0.062
## 1606639                                  + 13 -363.048 752.4  0.70  0.054
## 1610736                                  + 17 -358.975 752.5  0.77  0.053
## 1606640                                  + 16 -360.085 752.6  0.93  0.049
## 1577455                                  + 12 -364.205 752.7  0.97  0.048
## 1639407                                  + 13 -363.225 752.8  1.06  0.046
## 1573359                                  + 11 -365.297 752.8  1.12  0.044
## 1643503                                  + 14 -362.351 753.1  1.36  0.039
## 1639408                                  + 16 -360.321 753.1  1.40  0.038
## 1610223                                  + 13 -363.445 753.2  1.50  0.037
## 1577456                                  + 15 -361.449 753.3  1.60  0.035
## 1573360                                  + 14 -362.509 753.4  1.67  0.034
## 1643504                                  + 17 -359.449 753.4  1.72  0.033
## 1577960                                  + 15 -361.536 753.5  1.78  0.032
## 1573864                                  + 14 -362.563 753.5  1.78  0.032
## 1606127                                  + 12 -364.615 753.5  1.79  0.032
## 1672175                                  + 14 -362.666 753.7  1.99  0.029
## 1840111                                  + 14 -362.668 753.7  1.99  0.029
## Models ranked by AICc(x)
## Random terms (all models):
##   cond(1 | Replicate), cond(1 | LOC.ID)
```

Generalized linear mixed models for unmarked SHB: arrival pattern and relation to weather related factors

Unmarked beetles that are caught, have not been released, but have lived in the wild. For analysis of counts of unmarked beetles it does not make sense to include logNrelease and logDay.Since.REL as offsets. We also do not include Replicate, LogDist.Ms, Orientation and Dir as explanatory informatoin, as this information is quite irrelevant for free beetles.

```
#Records with REPLACED=1 are removed.
h.NoDye <- hives[hives$REPLACED != 1,
             c("SHBDYED", "SHBNODYE","D.M","D.F","logNrelease","logt.since.last.OBS2","logDist.Ms",
              "logDAY.Since.RELs","Dir","Orientation",
              "TEMPs","RHs","RAINs","WSs","WDdevs",
              "Replicate","LOC.ID")]
h.NoDye <- na.omit(h.NoDye)    # only cases without missing values are selected
dim(h.NoDye)
```

```
## [1] 591  17
```

```
#options(na.action = "na.fail")

glmmTMBoND <- glmmTMB(SHBNODYE ~ offset(logt.since.last.OBS2)
                             + TEMPs + RHs + RAINs + WSs
                             + (1 | LOC.ID),
                      family=nbinom2,
                      data=h.NoDye)
summary(glmmTMBoND)
```

```
##  Family: nbinom2  ( log )
```

```
## Formula:
## SHBNODYE ~ offset(logt.since.last.OBS2) + TEMPs + RHs + RAINs +
##     WSs + (1 | LOC.ID)
## Data: h.NoDye
##
##      AIC      BIC   logLik deviance df.resid
##   1047.8   1078.5   -516.9   1033.8      584
##
## Random effects:
##
## Conditional model:
##  Groups Name        Variance Std.Dev.
##  LOC.ID (Intercept) 0.4232   0.6505
## Number of obs: 591, groups:  LOC.ID, 76
##
## Dispersion parameter for nbinom2 family (): 0.705
##
## Conditional model:
##             Estimate Std. Error z value Pr(>|z|)
## (Intercept)  -8.9749     0.1381  -64.98  < 2e-16
## TEMPs         0.4748     0.1135    4.18 2.88e-05
## RHs           0.1794     0.1300    1.38  0.16737
## RAINs        -0.2994     0.1154   -2.60  0.00944
## WSs          -0.5719     0.1023   -5.59 2.29e-08
```

```r
# Analysis of deviance table using Wald-tests
Anova(glmmTMBoND)
```

```
## Analysis of Deviance Table (Type II Wald chisquare tests)
##
## Response: SHBNODYE
##         Chisq Df Pr(>Chisq)
## TEMPs 17.4942  1  2.882e-05
## RHs    1.9063  1   0.167373
## RAINs  6.7378  1   0.009439
## WSs   31.2314  1  2.290e-08
```

```r
# Analysis of deviance table using likelihood ratio tests
drop1(glmmTMBoND, test="Chisq")
```

```
## Single term deletions
##
## Model:
## SHBNODYE ~ offset(logt.since.last.OBS2) + TEMPs + RHs + RAINs +
##     WSs + (1 | LOC.ID)
##        Df    AIC     LRT  Pr(>Chi)
## <none>    1047.8
## TEMPs   1 1063.4 17.5323 2.825e-05
## RHs     1 1047.8  1.9182  0.166059
## RAINs   1 1052.7  6.8720  0.008756
## WSs     1 1075.2 29.3240 6.123e-08
```

```r
# Check model assumptions using functions from DHARMa library
simulationOutputND <- simulateResiduals(fittedModel = glmmTMBoND, n=1000)
plot(simulationOutputND)
```

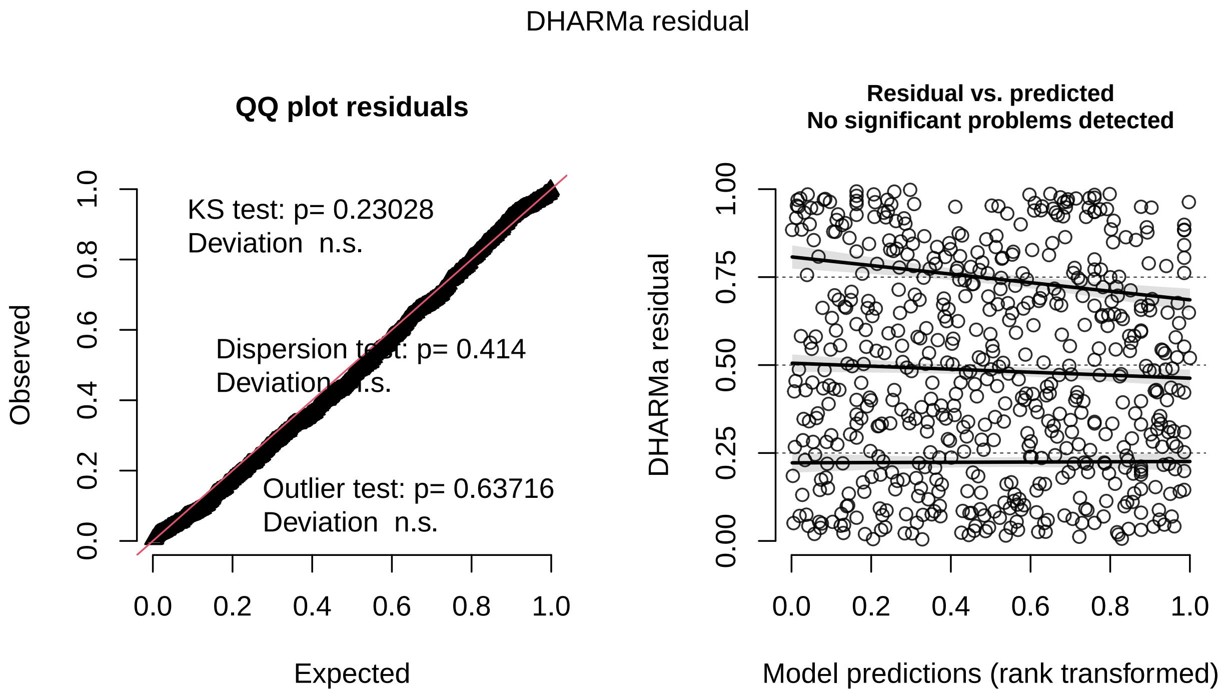


```
# All possible subsets using dredge function.
# We use previously obtained results.
#glmmTMBoND.dredge <- dredge(glmmTMBoND, trace=2)
#saveRDS(glmmTMBoND.dredge, "glmmTMBoND.dredge.rds")
glmmTMBoND.dredge <- readRDS("glmmTMBoND.dredge.rds")
# Print competing models at AICc value not further than 2 from best model.
# For model without sex we used 2.5, but that yields (too) many results for the model with sex.
subset(glmmTMBoND.dredge, delta < 2.5)
```

```
## Global model call: glmmTMB(formula = SHBNODYE ~ offset(logt.since.last.OBS2) + TEMPs +
##     RHs + RAINs + WSs + (1 | LOC.ID), data = h.NoDye, family = nbinom2,
##     ziformula = ~0, dispformula = ~1)
## ---
## Model selection table
##    cnd((Int)) dsp((Int)) cnd(RAI) cnd(RHs) cnd(TEM) cnd(WSs) df   logLik   AICc
## 13     -1.137          +                     0.3615  -0.3550  5 -511.919 1033.9
## 14     -1.138          +  0.05332            0.3765  -0.3611  6 -511.791 1035.7
## 15     -1.141          +          0.05402    0.3454  -0.3448  6 -511.798 1035.7
##    delta weight
## 13  0.00  0.551
## 14  1.78  0.226
## 15  1.80  0.224
## Models ranked by AICc(x)
## Random terms (all models):
##   cond(1 | LOC.ID)
```

Properties of individual SHB: weights

Weights have been measured of samples of prereleased beetles and of recaptured beetles.

Read beetles dataset into R. Make sure that some variables are factors. Log-transform some regressors and offset variables. Standardize quantitative regressors by substraction of the mean and dividing by the standard deviation.

```
beetles <- read.csv("Beetles.csv")

beetles$Source      <- as.factor(beetles$Source)
beetles$Sex         <- as.factor(beetles$Sex)
beetles$REPLICATE   <- as.factor(beetles$REPLICATE)
beetles$LOC.ID      <- as.factor(beetles$LOC.ID)
levels(beetles$Sex) <- c("M","F")  # 0=male, 1=female
```

Some descriptive statistics and visualizations of beetle weights

```
plot(Wgt ~ Sex, data=beetles)
```

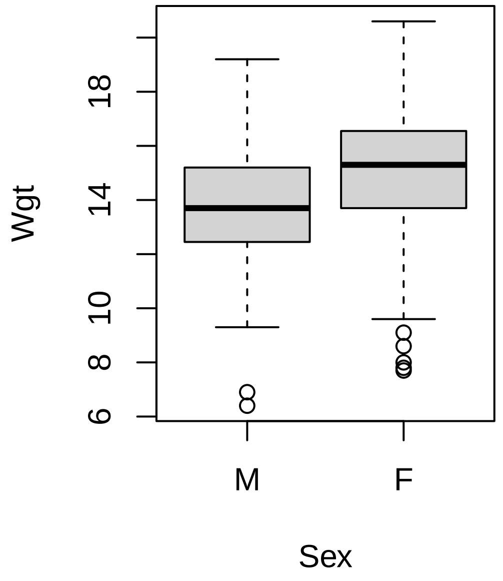


```
beetles$Sourcesex <- paste(beetles$Source, beetles$Sex, sep=".")
beetles$Sourcesex[is.na(beetles$Sex)] <- NA
table(beetles$Sourcesex)
```

```
##
## PREREL.F PREREL.M  RECAP.F  RECAP.M
##      185       45      125       38
```

```r
plot(Wgt ~ factor(Sourcesex), data=beetles)
```

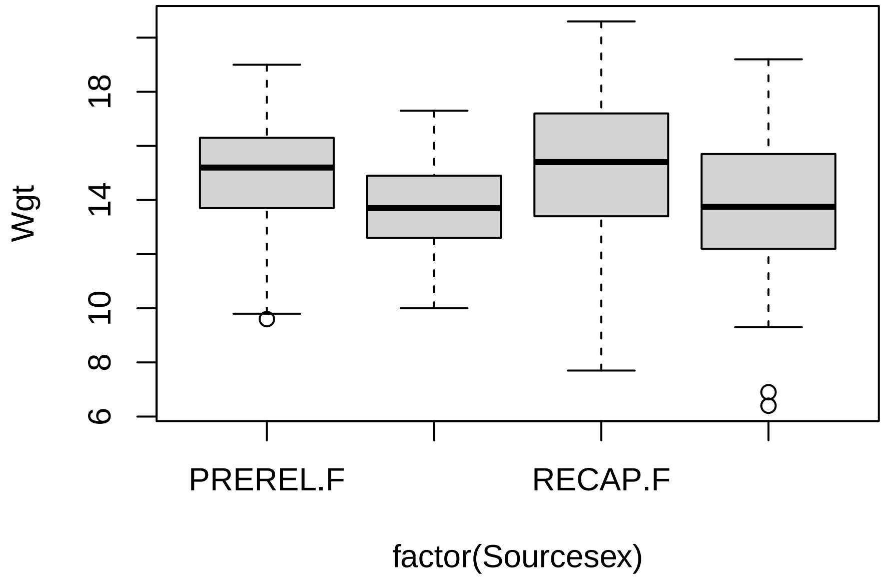


```r
# Pearson's chi-squared test for contingency table of
# prerelease/recaptured versus sex:
chisq.test(beetles$Source, beetles$Sex)
```

```
##
##  Pearson's Chi-squared test with Yates' continuity correction
##
## data:  beetles$Source and beetles$Sex
## X-squared = 0.59502, df = 1, p-value = 0.4405
```

```r
# Sex proportions prerelease and recaptured are not significantly different.
# So, sex ratio of arriving SHBs is not different from sex ratio of released SHBs.

beetles$SourceREP <- paste(beetles$Source, beetles$REPLICATE,sep=".")
beetles$SourceREP <- factor(beetles$SourceREP)
plot(Wgt ~ SourceREP, data=beetles, cex.axis=0.7, las=2, xlab=" ")
```

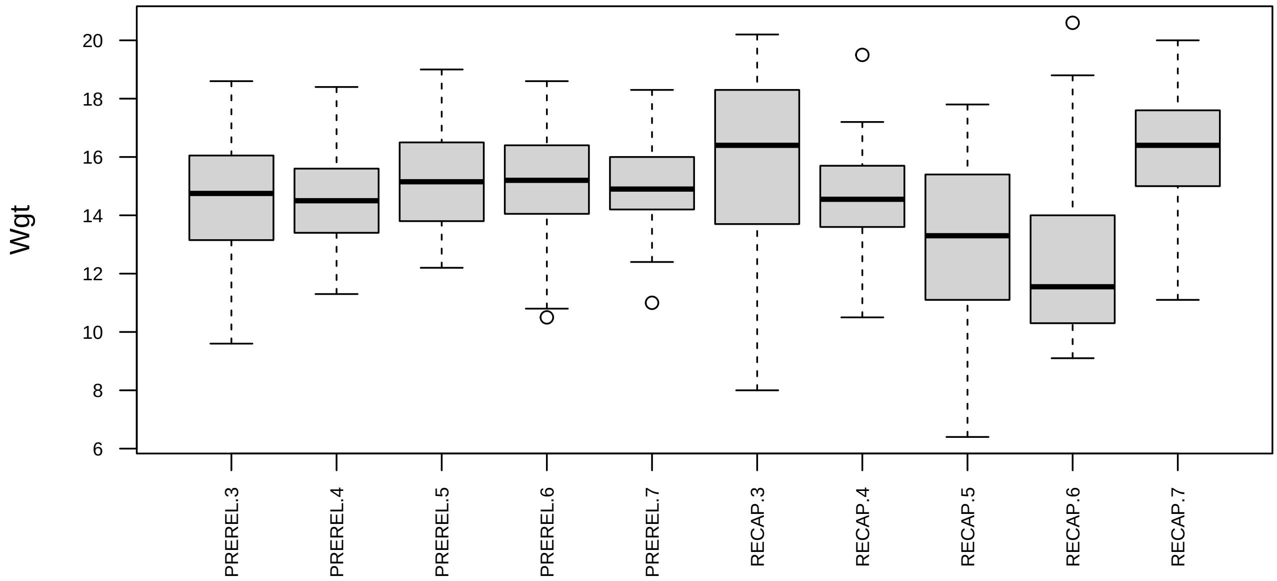


```
table(beetles$SourceREP)
```

```
##
## PREREL.3 PREREL.4 PREREL.5 PREREL.6 PREREL.7  RECAP.3  RECAP.4  RECAP.5
##       64       48       42       31       45       29       22       41
##  RECAP.6  RECAP.7
##       22       51
```

Individual SHB: beetle weights explained by source (prerelease / recaptured) and sexr

We have found weight variance to be quite different for sources and replicates. The weight variance is modeled alongside the weight means: we find higher variance for recaptured beetles and for replicates 3,5,6.

```
b <- beetles[, c("Wgt","Source","Sex","REPLICATE","LOC.ID")]
b <- na.omit(b)     # only cases without missing values are selected
dim(b)
```

```
## [1] 391   5
```

```
b <- droplevels(b)

glmmTMBoB <- glmmTMB(Wgt ~ Source + Sex + (1 | REPLICATE) + (1 | LOC.ID),
                          disp= ~ Source + REPLICATE,
                     contrasts=list(Source=contr.sum, Sex=contr.sum),
                     family=gaussian, data=b)
logLik(glmmTMBoB)
```

```
## 'log Lik.' -838.7108 (df=11)
```

```r
summary(glmmTMBoB)
```

```
##  Family: gaussian  ( identity )
## Formula:          Wgt ~ Source + Sex + (1 | REPLICATE) + (1 | LOC.ID)
## Dispersion:           ~Source + REPLICATE
## Data: b
##
##      AIC      BIC   logLik deviance df.resid
##   1699.4   1743.1   -838.7   1677.4      380
##
## Random effects:
##
## Conditional model:
##  Groups    Name        Variance  Std.Dev.
##  REPLICATE (Intercept) 1.727e-01 0.4155169
##  LOC.ID    (Intercept) 5.746e-09 0.0000758
##  Residual                     NA        NA
## Number of obs: 391, groups:  REPLICATE, 5; LOC.ID, 36
##
## Conditional model:
##             Estimate Std. Error z value Pr(>|z|)
## (Intercept)  14.3614     0.2401   59.81  < 2e-16
## Source1      -0.1028     0.1239   -0.83    0.407
## Sex1         -0.7671     0.1181   -6.50 8.27e-11
##
## Dispersion model:
##             Estimate Std. Error z value Pr(>|z|)
## (Intercept)  2.01439    0.15230  13.226  < 2e-16
## Source1     -0.48000    0.07871  -6.099 1.07e-09
## REPLICATE4  -0.92387    0.22697  -4.070 4.69e-05
## REPLICATE5  -0.21192    0.22630  -0.936    0.349
## REPLICATE6  -0.17901    0.25598  -0.699    0.484
## REPLICATE7  -1.07577    0.21065  -5.107 3.28e-07
```

```r
Anova(glmmTMBoB)
```

```
## Analysis of Deviance Table (Type II Wald chisquare tests)
##
## Response: Wgt
##          Chisq Df Pr(>Chisq)
## Source  0.6885  1     0.4067
## Sex    42.1930  1  8.269e-11
```

```r
drop1(glmmTMBoB, test="Chisq")
```

```
## Single term deletions
##
## Model:
## Wgt ~ Source + Sex + (1 | REPLICATE) + (1 | LOC.ID)
##        Df    AIC    LRT  Pr(>Chi)
## <none>    1699.4
## Source  1 1698.1  0.682     0.409
## Sex     1 1736.8 39.370 3.507e-10
```

```r
# DHARMa plot give problems while including random effects for LOC.ID.
# It is unclear why this happens.
# LOC.ID is not important, so we decided to remove it
# to be able to check DHARMa plots.
glmmTMBoB2 <- glmmTMB(Wgt ~ Source + Sex + (1 | REPLICATE),
                          disp= ~ Source + REPLICATE,
                      contrasts=list(Source=contr.sum, Sex=contr.sum),
                      family=gaussian, data=b)
logLik(glmmTMBoB2)
```

```
## 'log Lik.' -838.7108 (df=10)
```

```r
simulationOutput <- simulateResiduals(fittedModel = glmmTMBoB2, n=1000)
plot(simulationOutput)
```

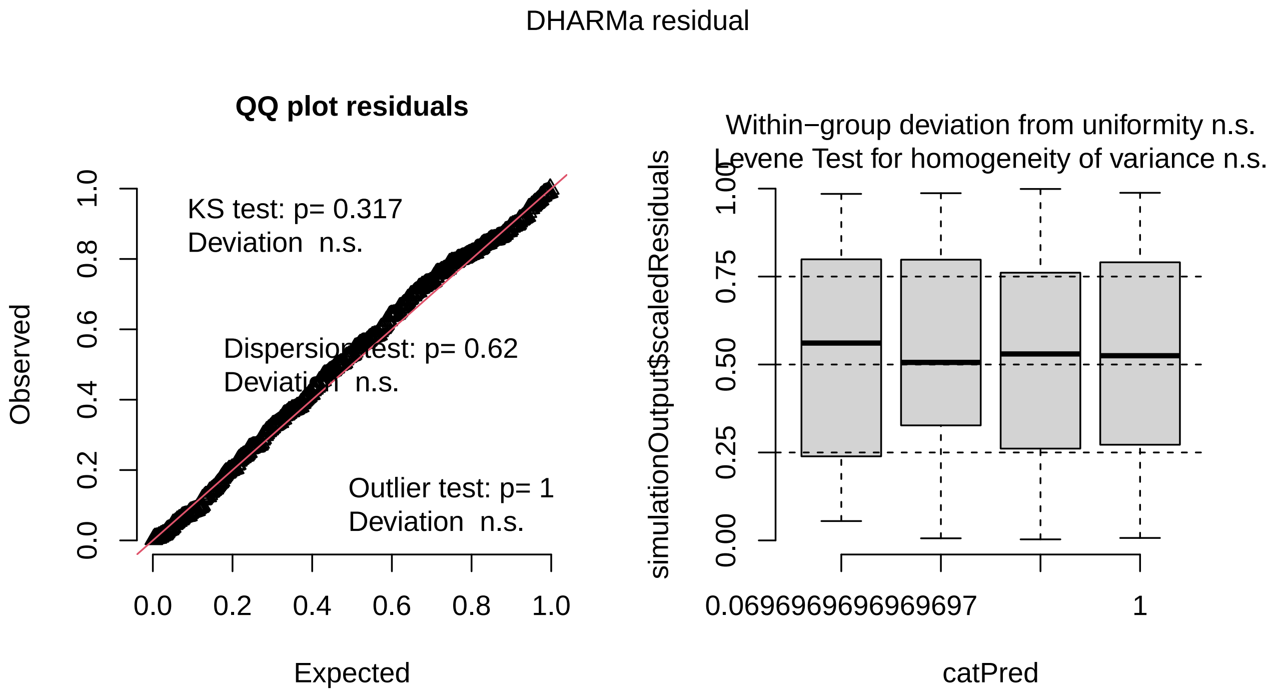

Individual SHB: beetle weights of recaptured explained by sex, location, time and weather characteristics

Select recaptured beetles. Standardize quantitative regressors.

```r
sel <- b$Source=="RECAP"

br <- beetles[sel, c("Wgt","Dist.M","DAY.Since.REL","Sex",
                     "TEMP","RH","RAIN","WS",
                     "REPLICATE","LOC.ID")]
br$TEMPs            <- scale(br$TEMP)
br$RHs              <- scale(br$RH)
br$RAINs            <- scale(br$RAIN)
br$WSs              <- scale(br$WS)
```

```r
br$logDist.M      <- log10(br$Dist.M+1)
br$logDist.Ms     <- scale(br$logDist.M)
br$DAY.Since.RELs <- scale(br$DAY.Since.REL)

br <- na.omit(br)    # only cases without missing values are selected
dim(br)
```

```
## [1] 153  17
```

```r
glmmTMBoBR <- glmmTMB(Wgt ~ logDist.Ms + DAY.Since.RELs + Sex +
                        + TEMPs + RHs + RAINs + WSs +
                        (1 | REPLICATE) + (1 | LOC.ID),
                      disp= ~ REPLICATE,
                      contrasts=list(Sex=contr.sum),
                      family=gaussian, data=br)
summary(glmmTMBoBR)
```

```
##  Family: gaussian  ( identity )
## Formula:
## Wgt ~ logDist.Ms + DAY.Since.RELs + Sex + +TEMPs + RHs + RAINs +
##     WSs + (1 | REPLICATE) + (1 | LOC.ID)
## Dispersion:           ~REPLICATE
## Data: br
##
##      AIC      BIC   logLik deviance df.resid
##    722.2    767.6   -346.1    692.2      138
##
## Random effects:
##
## Conditional model:
##  Groups    Name        Variance  Std.Dev.
##  REPLICATE (Intercept) 5.982e+00 2.4458749
##  LOC.ID    (Intercept) 7.889e-07 0.0008882
##  Residual                     NA        NA
## Number of obs: 153, groups:  REPLICATE, 5; LOC.ID, 32
##
## Conditional model:
##                 Estimate Std. Error z value Pr(>|z|)
## (Intercept)    14.286662   1.121399  12.740  < 2e-16
## logDist.Ms     -0.479303   0.284998  -1.682  0.09261
## DAY.Since.RELs  0.001473   0.357591   0.004  0.99671
## Sex1           -0.646456   0.222295  -2.908  0.00364
## TEMPs           0.331005   0.415522   0.797  0.42568
## RHs             1.247790   0.510857   2.443  0.01458
## RAINs           1.035413   0.238008   4.350 1.36e-05
## WSs            -0.135057   0.352217  -0.383  0.70139
##
## Dispersion model:
##             Estimate Std. Error z value Pr(>|z|)
## (Intercept)   2.4145     0.2824   8.548  < 2e-16
## REPLICATE4   -1.3328     0.4853  -2.747 0.006023
## REPLICATE5   -0.4106     0.3871  -1.061 0.288822
## REPLICATE6   -1.1855     0.4538  -2.612 0.008991
## REPLICATE7   -1.3559     0.3559  -3.810 0.000139
```

```r
Anova(glmmTMBoBR)
```

```
## Analysis of Deviance Table (Type II Wald chisquare tests)
##
## Response: Wgt
##                   Chisq Df Pr(>Chisq)
## logDist.Ms       2.8284  1   0.092612
## DAY.Since.RELs   0.0000  1   0.996713
## Sex              8.4570  1   0.003636
## TEMPs            0.6346  1   0.425683
## RHs              5.9660  1   0.014584
## RAINs           18.9254  1  1.359e-05
## WSs              0.1470  1   0.701387
```

```r
drop1(glmmTMBoBR, test="Chisq")
```

```
## Single term deletions
##
## Model:
## Wgt ~ logDist.Ms + DAY.Since.RELs + Sex + +TEMPs + RHs + RAINs +
##     WSs + (1 | REPLICATE) + (1 | LOC.ID)
##                 Df    AIC     LRT  Pr(>Chi)
## <none>             722.15
## logDist.Ms       1 722.98  2.8287 0.0925940
## DAY.Since.RELs   1 720.15  0.0000 0.9979985
## Sex              1 728.32  8.1661 0.0042680
## TEMPs            1 720.78  0.6295 0.4275396
## RHs              1 725.38  5.2287 0.0222168
## RAINs            1 732.44 12.2871 0.0004561
## WSs              1 720.29  0.1434 0.7049284
```

```r
simulationOutput <- simulateResiduals(fittedModel = glmmTMBoBR, n=1000)
plot(simulationOutput)
```

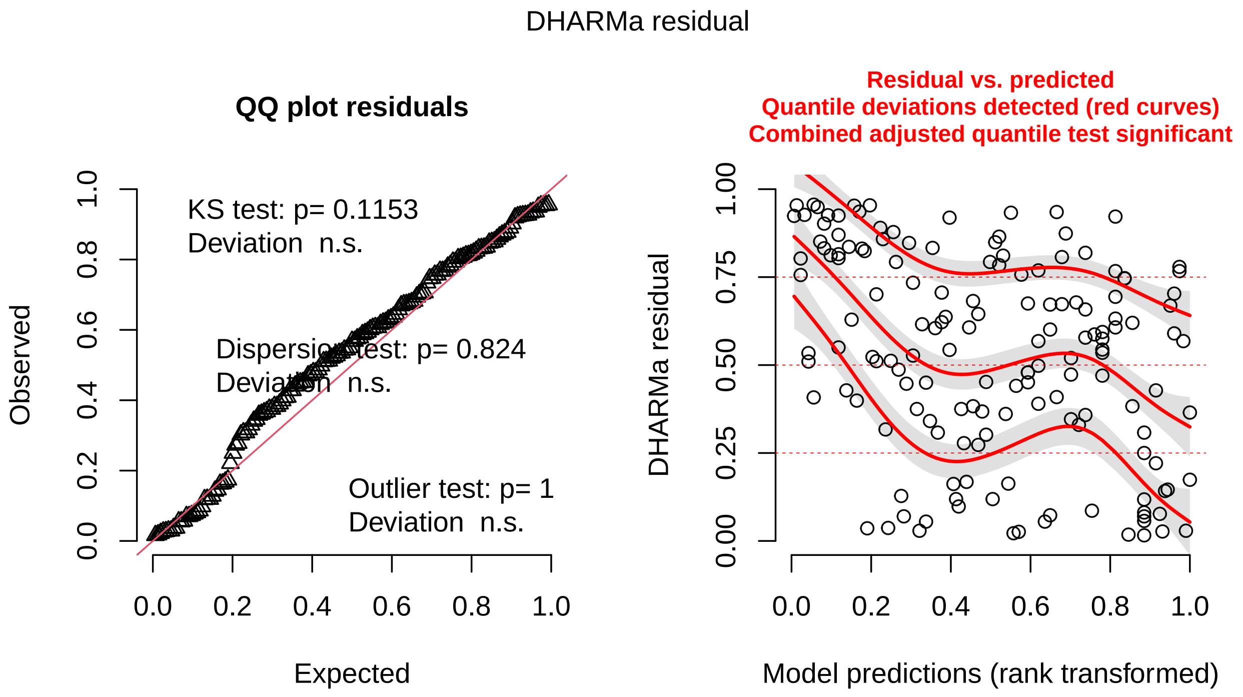


```
plot(residuals(glmmTMBoBR) ~ predict(glmmTMBoBR))
```

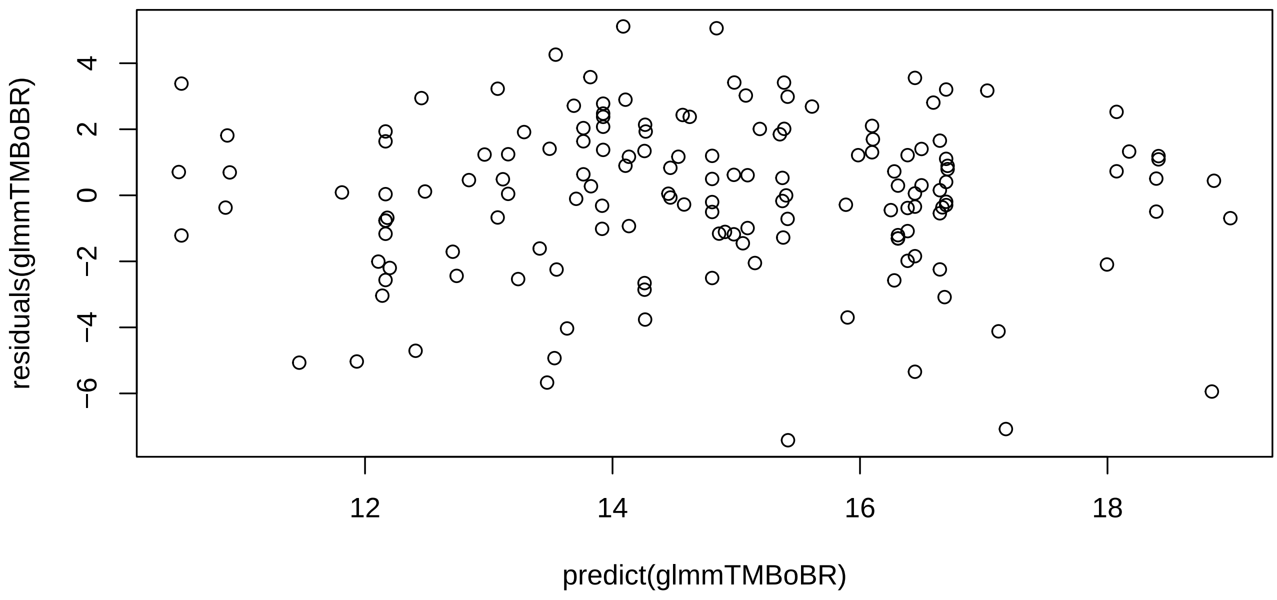

The QQ plot obtained with DHARMa shows acceptable residuals. However, in the residual versus prediction plot quantile deviations are observed. In the plot of ordinary residuals versus predicted values these patterns are less prominent.
